# A Precise Image-Based Tomato Leaf Disease Detection Approach Using PLPNet

**DOI:** 10.34133/plantphenomics.0042

**Published:** 2023-05-12

**Authors:** Zhiwen Tang, Xinyu He, Guoxiong Zhou, Aibin Chen, Yanfeng Wang, Liujun Li, Yahui Hu

**Affiliations:** ^1^ College of Computer and Information Engineering, Central South University of Forestry and Technology, Changsha 410004, Hunan, China.; ^2^ College of Bangor, Central South University of Forestry and Technology, Changsha 410004, Hunan, China.; ^3^ National University of Defense Technology, Changsha 410015, Hunan, China.; ^4^ Department of Soil and Water Systems, University of Idaho, Moscow, ID 83844, USA.; ^5^ Plant Protection Research Institute, Academy of Agricultural Sciences, Changsha 410125, Hunan, China.

## Abstract

Tomato leaf diseases have a significant impact on tomato cultivation modernization. Object detection is an important technique for disease prevention since it may collect reliable disease information. Tomato leaf diseases occur in a variety of environments, which can lead to intraclass variability and interclass similarity in the disease. Tomato plants are commonly planted in soil. When a disease occurs near the leaf’s edge, the soil backdrop in the image tends to interfere with the infected region. These problems can make tomato detection challenging. In this paper, we propose a precise image-based tomato leaf disease detection approach using PLPNet. First, a perceptual adaptive convolution module is proposed. It can effectively extract the disease’s defining characteristics. Second, a location reinforcement attention mechanism is proposed at the neck of the network. It suppresses the interference of the soil backdrop and prevents extraneous information from accessing the network’s feature fusion phase. Then, a proximity feature aggregation network with switchable atrous convolution and deconvolution is proposed by combining the mechanisms of secondary observation and feature consistency. The network solves the problem of disease interclass similarities. Finally, the experimental results show that PLPNet achieved 94.5% mean average precision with 50% thresholds (mAP50), 54.4% average recall (AR), and 25.45 frames per second (FPS) on a self-built dataset. The model is more accurate and specific for the detection of tomato leaf diseases than other popular detectors. Our proposed method may effectively improve conventional tomato leaf disease detection and provide modern tomato cultivation management with reference experience.

## Introduction

The tomato is native to South America and is widely cultivated in the southern and northern regions of China, where it is an economically important crop [1]. Most diseases of tomatoes start on the leaves and spread throughout the plant. Therefore, it is important to timely and accurately identify the disease types of leaves and to spray pesticides on the diseased areas to eliminate them. In the past, vegetable farmers usually checked for diseases with their own experience or asked relevant technicians and agricultural experts to guide them [2]. Such manual methods have many shortcomings: high workload, low detection efficiency, and vulnerability to visual fatigue. This renders the detection’s quality inconsistent. With the development of computers, especially the sharing of big data and the performance of computer hardware devices, the application of object detection technology to tomato production is the development trend of modern tomato cultivation [3,4].

Traditional machine learning approaches have been utilized successfully in the investigation of tomato leaf disease control. Deep learning is a significant advancement in this field and its powerful learning capability has enhanced the performance of neural networks [5]. It is a recently popular technique for visual image analysis. Among them, the deep learning-based object detection approach is the closest to traditional tomato leaf disease detection. The purpose is to identify the kind of disease while pinpointing the precise location of the diseased area. As a result, the approach may collect accurate information on tomatoes affected by pests and diseases, reducing producers’ profit loss [6].

Deep learning-based detection networks are generally divided into 2 groups. Faster Region-based Convolutional Neural Netwoks (R-CNN) [7] represents a two-stage network, while Single shot multibox detector (SSD) [8] and You only look once (YOLO) [9] represent a one-stage network. The primary distinctions between these 2 groups are as follows: the two-stage network must first generate candidate frames that might contain diseased areas before proceeding with the detection process, and one-stage networks, on the other hand, may directly use network features to predict the location and type of disease. The latter is more popular due to its faster speed. Specifically, the one-stage YOLO series is favored by researchers due to its exceptional extraction capability, high-speed detection architecture, and continual updates and maintenance.

In the early stages of the development of one-stage detectors, SSD and YOLO cannot satisfy the real requirements, and high-precision two-stage detectors still dominate practical applications. The later-introduced YOLOv3 [10] broke the dilemma, and its enormous speed advantage compensated for certain accuracy deficiencies. This has increased the popularity of one-stage detectors. Since then, frequent updates to the YOLO series have steadily enhanced detection precision and fostered the development of one-stage detectors. Simultaneously, the rapid growth of deep learning technology has led to the emergence of numerous outstanding tricks. On this foundation, YOLOv4 [11] by Bochkovskiy et al. combines numerous outstanding ideas and is a rapid and simple-to-train object detector. In the end, YOLOv3 was optimized in terms of data processing, training strategy, backbone network, pyramid structure, activation function, and loss function after extensive testing on some regularly used tricks. Compared to other detectors of the period, YOLOv4 is the most advanced real-time one-stage detector and can be readily implemented on a single GPU. The succeeding YOLOv5 [12] offers a multi-size model while optimizing even more. This improvement greatly increases the YOLO detector’s flexibility to diverse application conditions. Ge et al. [13] proposed YOLOX in 2021, based on YOLOv3. During the network’s detection phase, YOLOX employs decouple head to enhance the expressive capacity of the detection head and accelerate the network’s convergence. YOLOX employs an anchor-free manner for the first time, which decreases the number of anchor frame calculations by two-thirds compared to the series version. The SimOTA strategy evaluates the optimal solution for label assignment from a global perspective during the label assignment phase. These modifications greatly increase the accuracy of detection. In general, YOLOX is superior to previous versions, pushing the YOLO series to new heights in terms of speed and precision.

In recent years, several researchers have utilized the YOLO series for tomato disease detection, which offers a reference for our study. For example, in 2020, Chakravarthy and Raman [14] used YOLOv3-SPP to detect early blight of tomatoes and proved the practicality of using this detector. In 2021, Wang et al. [15] combined inverse residual blocks with the YOLOv3-tiny architecture. This approach reduces network information transmission loss, improves the feature extraction process, and achieves a good speed in tomato disease detection. In 2021, Wang and Liu [16] proposed a network for detecting tomato diseases in natural environments. They replaced the YOLOv3 source backbone network with MobileNetv2 to reduce the number of parameters. Then, they incorporated both high-level and low-level features into feature maps together via a multi-scale module and enhanced the feature maps with a channel attention module. The network achieves an average accuracy of 93.4% and an inference time of 0.022 s on the self-built tomato botrytis. In 2022, Liu et al. [17] proposed a YOLOv4-based network for tomato pests’ detection. They added a triple attention mechanism to the network to boost the weight of effective feature channels. They also used focal loss in the network’s prediction process to improve the network’s perception of tiny objects. K-means++ was utilized to improve the original anchor frame method based on the image features of tomato pests. The final experiment demonstrates that the method enhanced mAP by 6.3% while maintaining speed. In 2022, Qi et al. [18] attempted to add the Squeeze-and-Excitation block into the YOLOv5 model for key feature extraction. Finally, 94.10% of mAP50 and 75.98% of mAP were acquired from the greenhouse tomato disease picture dataset, but the FPS was only 19.75.

The above researches have shown good results and demonstrated the viability of the YOLO series for the detection of tomato leaf diseases. However, their researches focused primarily on problems such as detecting a specific disease or detecting tiny objects. They did not study the association between representative disease characteristics and the network, nor did they analyze the impact of natural background on detection. More research in the field of tomato leaf disease detection is demanded to explore deeper disease features and improve the professionalism of the network. Thus, we chose YOLOX as the baseline network. In consideration of the limited computational resources, in this paper, the YOLOX-S version with a small number of parameters is chosen to explore the feasibility of enhancing tomato leaf disease detection using deep learning techniques.

**Fig. 1.**
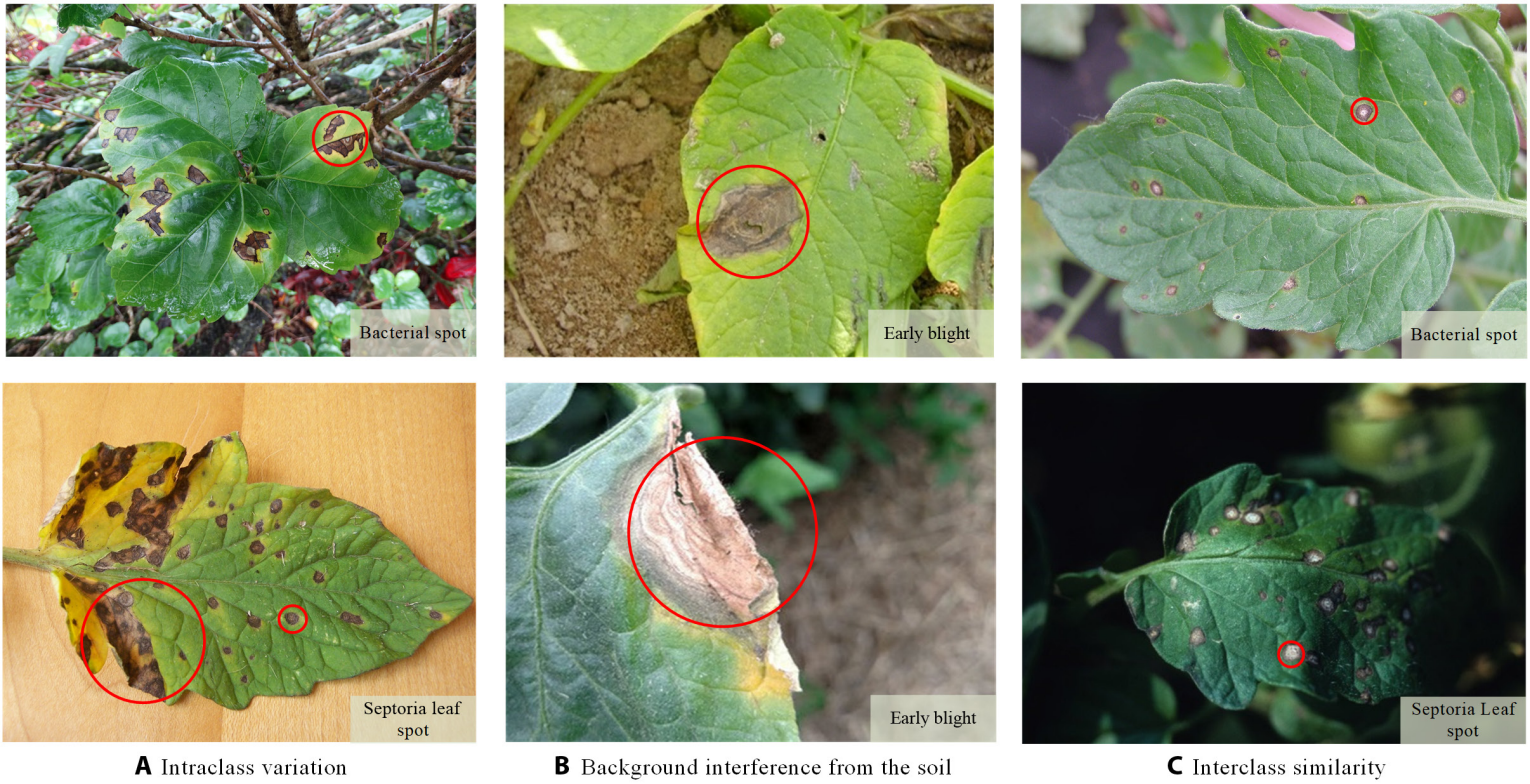
Problems with tomato leaf disease detection.

Several issues must be addressed in the existing tomato leaf disease detection procedure (as shown in Fig. 1): (a) Intraclass variation. Because different lesions form in diverse surroundings, the same disease frequently shows various pathological states, such as different colors, textures, and shapes. Previous detection networks frequently overlooked this hybrid feature. (b) Background interference from the soil. In natural environments, the soil environment of tomatoes frequently shares color features with a number of diseases. The information reference of the adjoining healthy section lowers the interference of the backdrop when the disease spot is in the center of the leaf. However, when lesions occur near the leaf’s edge, such reference information is absent. In such situations, the interference of the soil backdrop frequently leads to detection network misjudgment. (c) Interclass similarity. Numerous studies have investigated the fundamental practicality of employing biomorph (shape and structure) to differentiate separate categories [19]; however, this is often effective when the class difference is more marked. Certain types of diseases have similar color, texture, form, and other characteristics. These slight variations make detection difficult.

Some researches give ideas for our solutions to the aforementioned issues. For example, (a) Li et al. [20] proposed a wide and deep feature extraction block (WDBlock) for the tomato leaf disease image generation task. This module deepens and widens the number of convolutions through 2 routes, thus capturing the disease’s deep and global features, respectively. This structure enriched the feature information of the backbone network and enhanced the image generation quality. The widening route contains convolution kernels of various sizes, which is able to collect multi-size disease feature. For diseases with multiple pathological states, notably form and size variation, it is difficult for a single-size convolution to account for the various disease characteristics. However, multi-scale convolutions may pay attention to different disease characteristics from different viewpoints, allowing the same feature layer to hold more disease information. (b) In the categorization of butterfly, Li et al. [21] employed a cross-attention mechanism (CAM) to overcome the problem of natural background interference. The mechanism extracts feature in the vertical and horizontal directions, based on 2 weight distribution algorithms. This method effectively suppresses the expression of redundant information in the background while having a favorable influence on the identification of butterfly pictures with various spatial distributions. This demonstrates that attention mechanisms can efficiently filter out background noise. In addition, unlike conventional attention mechanism, which only considers a single direction, the cross structure perceives the information properties of the target region in the vertical and horizontal directions independently and is more sensitive to target location. These strengths provide a reference for solving the issue of confusing edge disease with soil background in the network. (c) To enhance the performance of long-distance smoke detection, Zhan et al. [22] proposed a recursive feature pyramid with deconvolution and dilated convolution (RDDFPN). The pyramid structure employs a 2-layer recursive method to execute 2 fusion processes on the backbone network’s feature information. This method of recurrent fusing enhances the detector’s fusion efficiency and detection precision. For comparable disease characteristics with small variations, the secondary fusion method can reduce the probability of omitting critical feature information during the initial fusion. This gives a paradigm for solving the problem of disease similarity. However, directly recursing twice on the full pyramid structure will result in an enormous number of parameters. In addition, because the disease target is not as tiny as the remote smoke, this will enable the network to gather a huge number of similar features, resulting in a process known as overfitting. Therefore, we use a more microscopic degree of convolution for this idea.

The following is a summary of this paper’s contributions.

1. For the object detection network to obtain rich tomato leaf disease characteristics, we built a precisely labeled dataset containing 5 types of diseases. Classification labels and precise bounding boxes are applied to label all disease areas in the dataset.

2. To solve tomato leaf disease detection problems, we propose a precise image-based tomato leaf disease detection approach using PLPNet. The design is as follows:

a. A perceptual adaptive convolution (PAC) module is proposed. This module may adjust the weight of different convolution kernels based on the characteristics of the disease area to extract more details about the disease. This improves the network’s capacity to link information surrounding the target and has a beneficial effect on diseases with multiple traits.

b. A location reinforcement attention mechanism (LRAM) with integrated spatial and channel information is proposed. This mechanism assigns more weight to the disease’s target area by using horizontal and vertical feature vectors. It can effectively detect edge diseases as well as filter background information elements such as soil that may interfere.

c. A proximity feature aggregation network with switchable atrous convolution and deconvolution (SD-PFAN) is proposed. First, switchable atrous convolution (SAC) is used to multiplex micro feature information. Then, deconvolution inverse maps the low-level information to the high-level feature layer. Finally, the proximity feature aggregation network fuses the shallow visual and deep semantic information of the feature maps. This structure efficiently incorporates minor distinguishing features, which greatly enhances disease detection performance.

3. The PLPNet proposed in this paper achieved 94.5% mAP50 and 25.84 FPS on a self-built dataset. The approach effectively extracts tomato leaf disease characteristics with complex textures and irregular shapes. It recognizes and detects tomato leaf diseases with similar pathological status. In general, this approach can detect tomato leaf diseases quickly and precisely, and it may serve as a reference for the control of tomato diseases in large-scale tomato production.

## Materials and Methods

### Data acquisition

Because the tomato leaf disease detection dataset is the foundation of the study, we chose 5 categories of tomato leaf diseases, the characteristics and numbers of which are shown in Table 1. The dataset in this paper is composed mostly of 2 parts, one of which was filtered from PlantVillage [23]. Because this dataset was not maintained and updated, there are drawbacks such as fuzzy photos and misclassification. With the guidance of our professional team, we removed the following components from this dataset in order for the network model to extract effective features: (a) full leaf withering, (b) fuzzy pixels, (c) inadequate light, and (d) misclassification; a total of 3,524 images were finally obtained. Furthermore, 1,909 images of tomato leaf diseases from the Internet were filtered for the second part by using processing criteria mentioned above.

**Table 1.** The number and characteristics of tomato leaves’ images.

Category	Example	Characteristics	Number (before/after)	Proportion (before/after)
Healthy	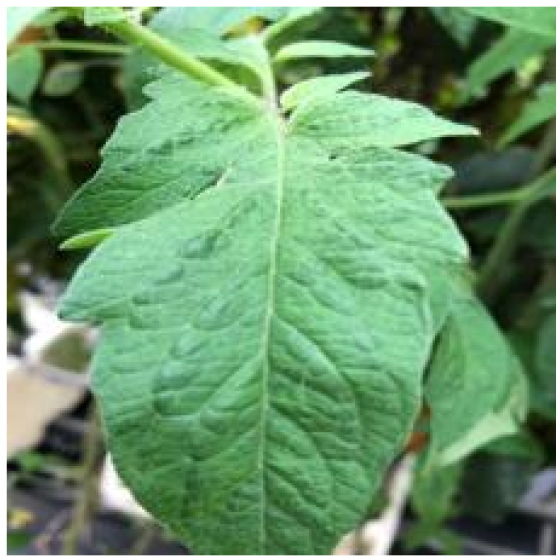	The leaves are grassy green in color and texture, with no disease spots on the surface and distinct veins.	–	–
Bacterial spot	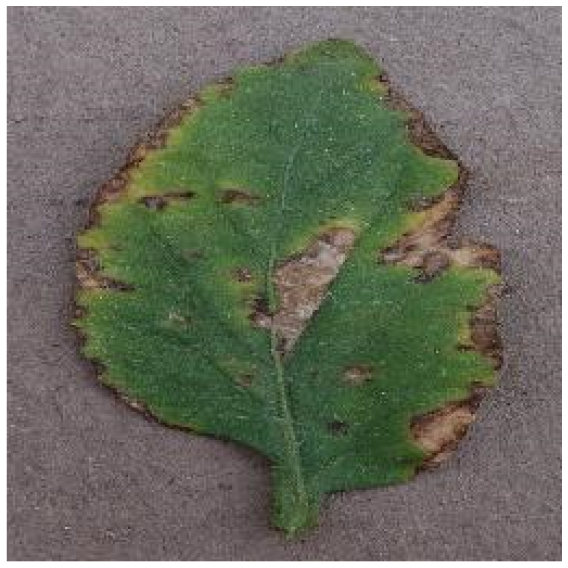	The disease mostly damages the margins of the leaves, presenting gray-brown patches with a yellow halo surrounding them.	1,112/2,708	20.47%/19.92%
Early blight	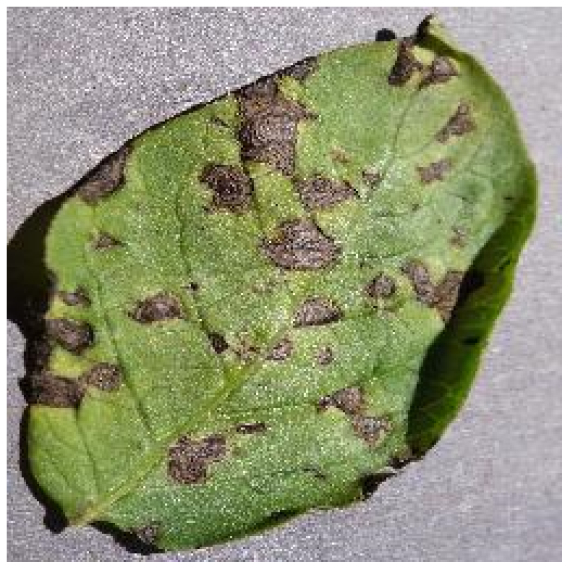	The leaves generate little dark brown sub-circular spots, which are often linked to form larger irregular patches.	841/2,733	15.48%/20.10%
Late blight	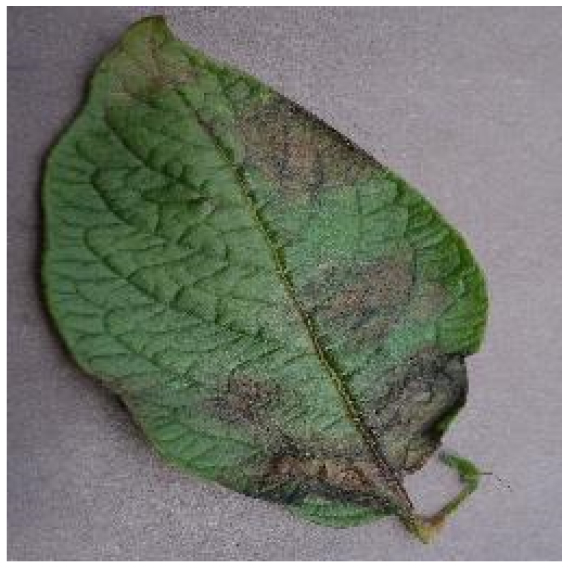	The diseased area is a large area of dark brown water-soaked patchiness.	1,202/2,705	22.12%/19.89%
Leaf mold	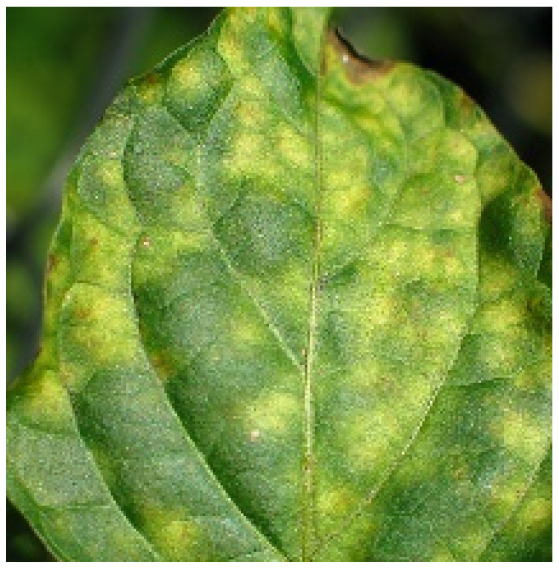	The spots are yellow-green in hue and frequently cover a large area, filling nearly the whole leaf.	881/2,758	16.22%/20.28%
Septoria leaf spot	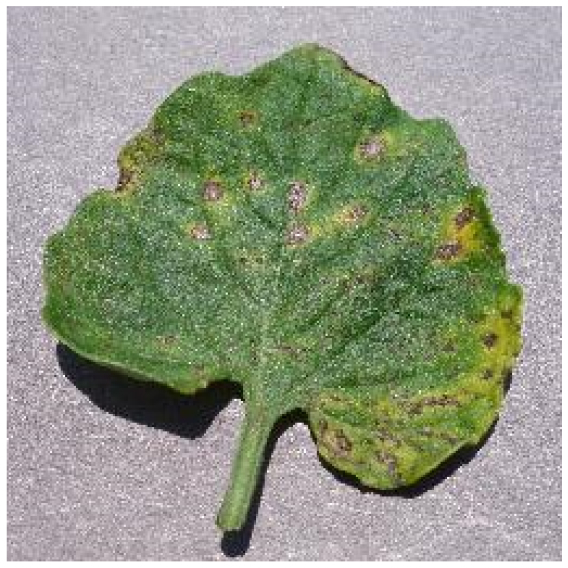	The spots are gray-brown with a yellow halo, and the scale is often tiny, resembling a fish-eye appearance.	1,397/2,693	25.71%/19.81%

We analyzed and chose the characteristics of the following 5 tomato leaf diseases for the network model to detect: (a) bacterial spot, (b) early blight, (c) late blight, (d) leaf mold, and (e) septoria leaf spot. Tomato leaves that are healthy are normally grass green in color, with a disease-free surface and visible veins. We observed that when bacterial spot and septoria leaf spot damage the leaves, the disease area is generally gray-brown with a yellow halo surrounding the spot. They differ in that the latter are typically smaller in size, while the former is from various sizes and contain marginally susceptible areas. If the network just has one size, it will most likely be unable to deal with such a broad range of feature information, resulting in lower detection accuracy for both categories. Tomato early blight and late blight have a similar color texture, both being dark brown. However, there is a marked disparity in their distribution. Early blight patches are frequently connected together to form large irregular spots, but late blight is more dispersed and covers a broader area. Leaf mold is the most distinct of these 5 types of diseases, with spots that are predominantly yellow-green and typically more widely spread throughout the leaf.

There are numerous similarities among these 5 disease categories. For example, early blight and late blight have similar textures, and the spots are generally brown in color and nearly round in shape. However, certain diseases present multiple characteristics, such as septoria leaf spot, which has a brown core surrounded by a yellow halo. The bacterial spot is another example, as it contains edge objects that are more likely to be mistaken with comparable backdrops. Consequently, studying the detection of these 5 tomato leaf diseases serves as a benchmark for the investigation of additional tomato leaf diseases.

### Data processing

**Fig. 2.**
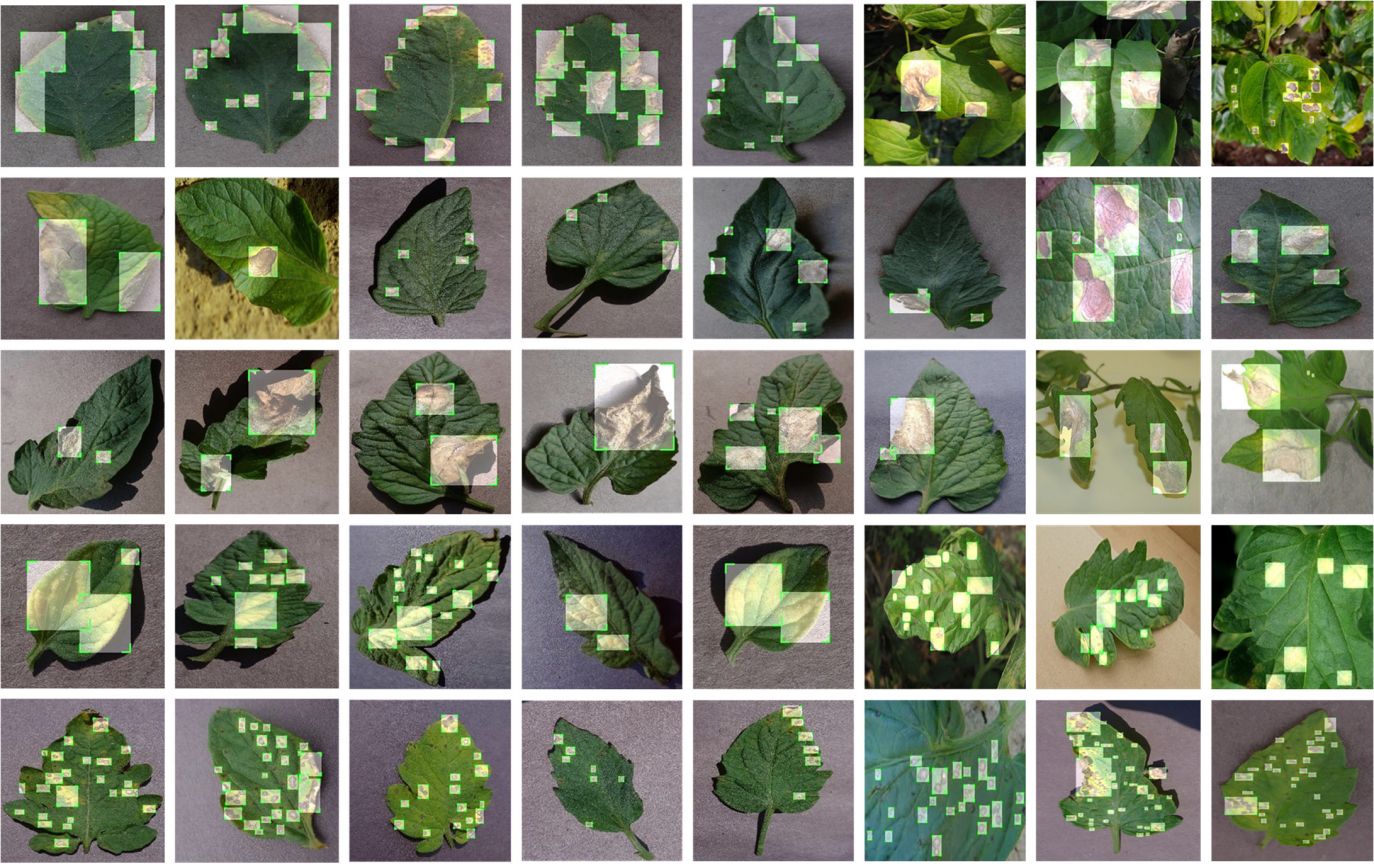
Examples of partial image labels.

Because deep neural network models require a large number of images to extract effective features and avoid issues like overfitting, the original dataset must be augmented. This paper’s data augmentation includes (a) horizontal or vertical flip, (b) Gaussian noise or Gaussian blur, and (c) brightness light or dark changes to simulate shifts in sunshine. As indicated in Table 1, to minimize the influence of data distribution on network training, the number of augmented categories should be kept as balanced as feasible. Table 2 shows the image augmentation results for the septoria leaf spot as an example. This dataset must also be labeled with the corresponding bounding boxes for the object detection task. With the assistance of a team of agricultural specialists, we identified all lesions in all images by category and carefully chose all pixels in the lesion area. This enables the bounding box to accurately label the disease’s category and region information. Each image’s annotations are saved in XML format and the portion of the data containing annotations is presented in Fig. 2.

**Table 2.** Data augmentation type.

Horizontal flip	Gaussian blur	20% brighter
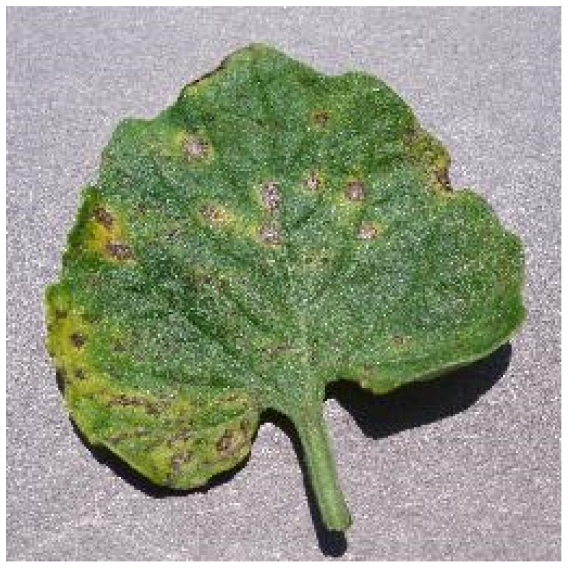	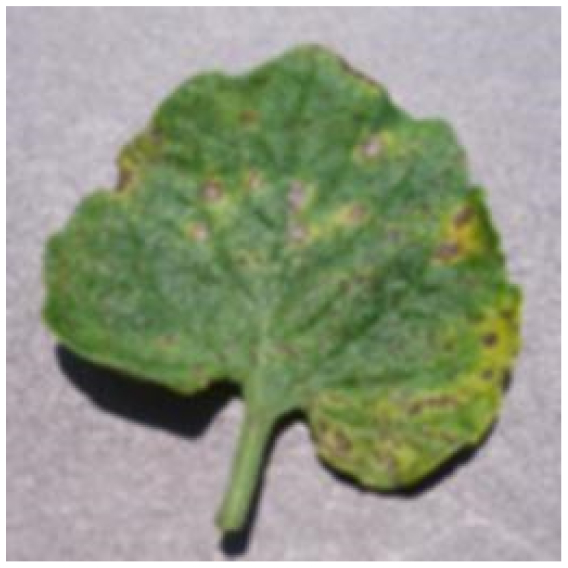	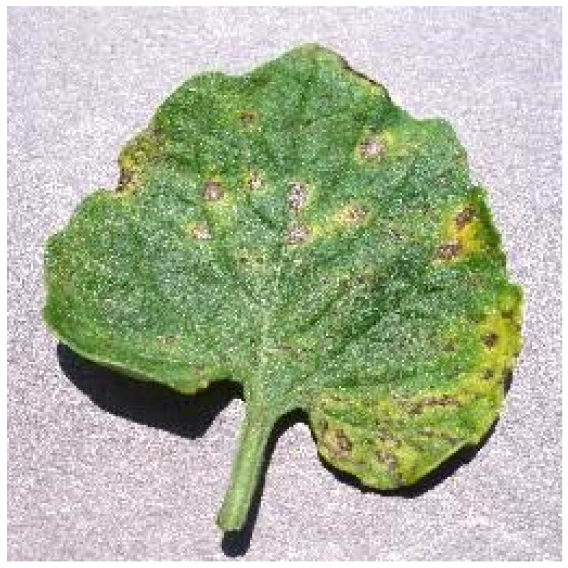
Vertical flip	Gaussian noise	20% less brightness
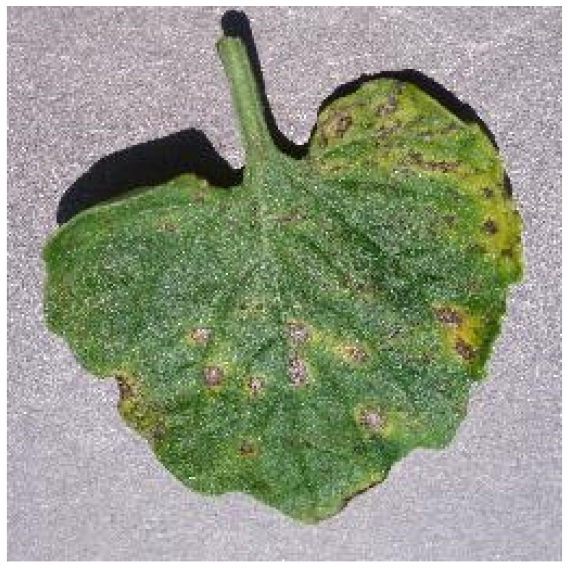	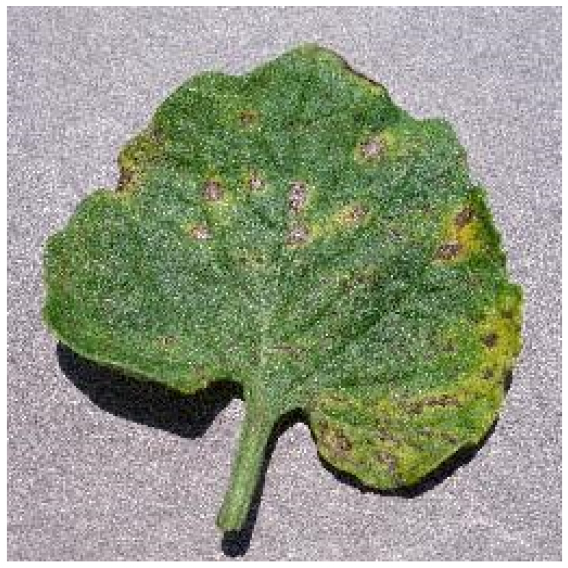	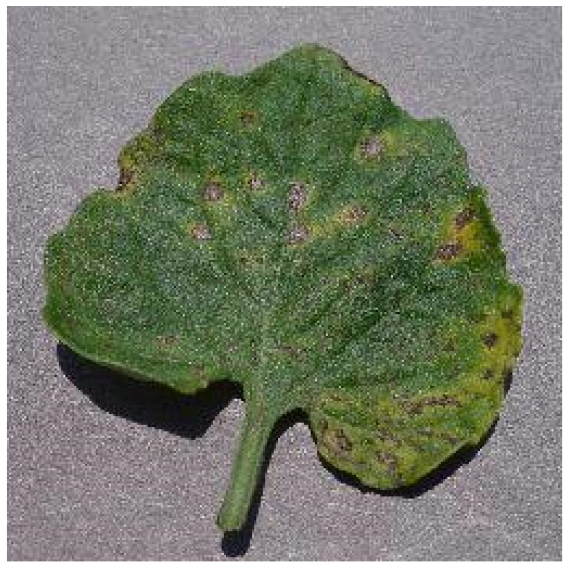

### PLPNet

**Fig. 3.**
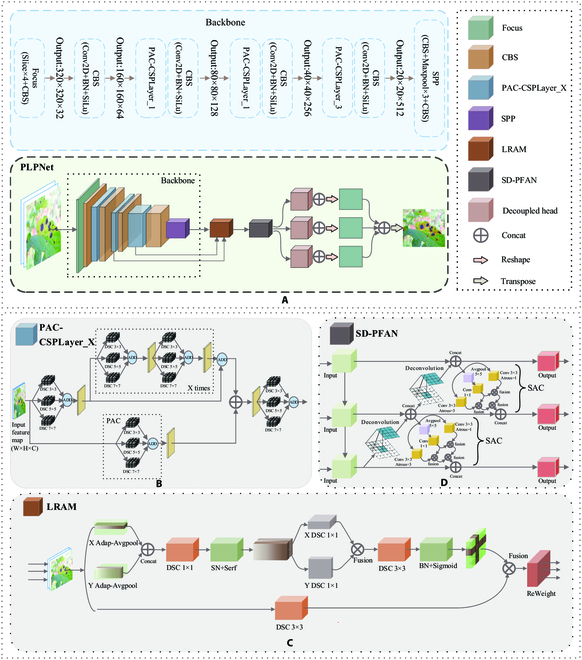
The structure of PLPNet.

In this paper, a YOLOX-S-based object detection approach for tomato leaf diseases, PLPNet, is proposed for the characteristics of tomato leaf diseases. Its network model structure is shown in Fig. 3A. During the feature extraction process, we propose a PAC module and replace all of the standard CSPLayer convolutions in the backbone network. The PAC-CSPLayer is utilized to extract richer disease characteristics and enhance the network’s global sensing capability. We propose an LRAM before the feature information enters the fusion process and add it to the link between the backbone network and the pyramid structure. It reduces the chance of mistake generated by unnecessary information entering the feature fusion process. SD-PFAN is proposed in this paper during the feature fusion process. This structure, as compared to the original path aggregation network (PANet), may better fuse the feature information of different backbone network layers. It provides better classification and detection suggestions for the network’s future detection process.

#### Perceptual adaptive convolution

Tomato leaf diseases often include a wide range of scales, such as septoria leaf spot in tiny sizes, late blight huge areas in big sizes, and early blight medium sizes of brown spots. However, there are also different sizes for the same disease. Furthermore, the texture of certain diseases varies, such as the septoria leaf spot, which has distinct colors on the exterior and interior of the spot. Although normal 3 × 3 convolution has been able to capture lesion characteristics of regular sizes, a fixed convolutional layer has a restricted mapping range. This could not completely acquire the global information of large-scale diseases, which impacts the positioning and precision of detection. In addition, all sample points in the feature map are treated equally by the fixed convolution filter due to the parameter sharing of the convolution operation. Different sample points have varying signal-to-noise ratios, limiting the model’s capacity to reflect the properties of tomato leaf diseases. Therefore, we attempt to add more convolution sizes to obtain diverse disease characteristics.

As stated in the Introduction, Li et al. illustrate that deeper and more extensive convolution numbers can enhance disease information. This idea originates in the Inception modules of GoogleNet [24]. They demonstrated the possibility of enhancing visual neural networks with dense parallel architectures. Compared to narrower networks, these architectures provide remarkable quality improvements with minimal increases in computing overhead. Based on the aforementioned application and theoretical study, this paper proposes a PAC module, as seen in Fig. 3B, for the diverse characteristics of tomato leaf diseases. This module gathers and maintains as many of the same diseased area’s various characteristics as feasible using parallel convolution operations at 3 scales. This can lower the likelihood of the network misdetecting different features of the same disease as other categories, while improving the detection accuracy of the network.

Similar to Inception modules, PAC has 3 different convolution kernels to enhance the expression of single-layer characteristics. The distinction is that PAC does not contain Maxpool. Due to the fact that YOLOX employs SPP components (multiple Maxpool parallel structures) at the network’s end, the pooling process is unnecessary. In addition, in the process of convolutional merging, Inception uses 1 × 1 convolutional dimensionality reduction and depth channel stacking. These options alter the channel’s size. Nevertheless, channel features often comprise color and texture traits for tomato leaf disease. Changing the channel’s dimensions will result in the loss of characteristic information, which is not favorable to preserving the disease’s complete features. Consequently, PAC’s multi-core convolution retains the same number of input and output channels. The final stacking process is performed by adding elements, which is also inexpensive and efficient. The operation process of PAC will be introduced in detail below.

When adjusting convolution kernel sizes, a larger convolution kernel provides a wider field of vision. However, it also increases the number of parameters, resulting in higher training costs. To solve this problem, we construct convolution layers using depthwise separable convolution (DSC) [25] instead of standard convolution. PAC firstly creates new feature maps with richer features by employing DSCs with kernel sizes of 3 × 3, 5 × 5, and 7 × 7 for the input tomato leaf disease images, respectively. This results in the new feature maps having large, medium, and small receptive fields respectively, and the number of channels is equal to the original feature map. This can contain variable-sized feature information. PAC then superimposes and combines the feature maps to enhance the informational richness of disease characteristics. Due to the presence of DSC, these operations do not enhance the depth or width of the network, but can improve expressiveness by combining multiple convolution kernels.

In the figure, the rectangle denotes the feature map, the gray-black cube denotes the DSC of different size convolution kernels, W and H denote the width and height of the feature map, respectively, C denotes dimensionality, ADD denotes pixel summation, and X denotes the number of repetitions in the box section. When the network begins backpropagation, PAC adjusts the weights of different convolution kernels based on the prediction error. This decreases the computational bias of the network’s predictions with respect to the predicted values. The more suitable convolutional kernel size will play a more important portion in this process. In contrast, the proportion of mismatched convolutional kernel sizes will drop. The inner weights of PAC are approximated toward the optimal convolutional kernel as the training epoch increases. As a result, the convolutional kernel takes priority, with the remaining portions serving as information supplements. This design is capable of capturing complicated characteristics of tomato leaf diseases more effectively than the standard convolutional fixed extraction capacity. Furthermore, the ADD method required only a few mathematical operations, does not introduce training parameters, and provides additional information while maintaining the number of network parameters constant. The Effectiveness of PAC section presents an experimental analysis of PAC’s effectiveness.

#### Location reinforcement attention mechanism

In the research of tomato leaf disease detection, soil background interference often has a greater influence on the detection network. In approaches for deep learning, the attention mechanism may be used to suppress such background noise and guide the network’s emphasis on crucial target regions. Attention mechanisms have been extensively studied and have been demonstrated to considerably enhance the performance of visual neural networks [26]. Early SE [27] increases attention to the channel dimension, modeling the significance of each feature channel. Then, it assigns different weights to each feature based on the usefulness of each channel, suppressing or emphasizing the expression of feature channels. This not only enhances the network’s performance but also is robust against noisy inputs. The subsequent convolutional block attention module (CBAM) [28] introduced spatial attention based on SE, demonstrating that the combination of both can result in enhanced performance. Unlike the feature attention of channel attention, spatial attention focuses on the target’s position information and can supplement it. This refines the feature representation more.

For marginal diseases, such as bacterial spot, the disease characteristics typically resemble the background soil and border each other spatially. This usually causes the detection network to mistake background soil for a disease. Currently, if merely channel attention (such as SE) or spatial attention is utilized, the results are usually poor owing to a lack of localization or feature information. This difficulty is effectively solved by CBAM’s comprehensive information. As stated in the Introduction, a cross structure might increase the sensitivity of target areas to localization. Coordinate attention (CA) [29] has a similar structure and also contains channel and spatial data. It involves crossover structures based on channel and spatial attention. This allows CA to simulate internal channel properties while capturing long-distance positional information dependency. This has substantial significance for the detection of edge diseases near to soil.

Taking into account the complexity of disease characteristics and the distribution of training batches, we optimize the nonlinear unit and normalizing layer of CA. This enables CA to serve as the attention in a more comprehensive lesion characteristic. Moreover, the new normalizing layer may eliminate the issue of class imbalance in the training batch. In addition, the attention mechanism cuts the input feature channel and performs the calculation on a smaller feature scale. Although this can greatly minimize computing overhead, some feature information will be lost (as described in the Perceptual adaptive convolution section). Before its output attention map enters feature fusion, we add a layer of 3 × 3 convolution on the decreased size, which increases the attention region’s feature representation. Although this increases the number of parameters, it improves the efficiency of the subsequent fusion process and enhances the accuracy of detection. The modified CA is named as LRAM in Fig. 3C.

The LRAM is composed of 2 primary parts:

1. Generate the direction-aware feature map *F_D_*. We horizontally and vertically decompose the input feature map along the channel direction to generate a pair of crossing tensors. Following that, adaptive averaging pooling is employed to acquire global feature information in both directions. Finally, we concatenate the feature information along the direction of the channel, embed the location information, and yield a feature map with direction awareness. In the middle convolutional layer, the feature representation is refined.

Considering the influence of training batch and the complexity of disease characteristics, we replace standard batch normalization (BN) [30] with switch normalization (SN) [31]. Compared to BN, SN is more robust in a wide variety of batch sizes and performs well even with tiny batches. SN can also adapt to different network architectures end-to-end and does not require batches as training hyperparameters. This eliminates variations in the distribution of data produced by different batch settings. These strengths are compatible with limited computational resources. For the nonlinear mapping layer, we substitute the standard ReLU [32] with the Serf [33] function to increase the model’s nonlinear capacity. It avoids discarding ReLU's negative information and maps all information thoroughly. This enables LRAM to conduct attention calculations on more comprehensive lesion features, hence enhancing the accuracy of object region detection.

The formula for Serf is as follows:

$$f\left(x\right)=x\cdot \mathrm{erf}\left(\text{ln}\left(1+e^{x}\right)\right)$$ (1)

2. Update the weights of the feature map. We separate the feature map into 2 independent tensors along the channel dimension and conduct feature improvement separately after embedding the location information into the channel features. We employ a more efficient multiplication operation to enhance the vital part of the feature information to minimize the interference of irrelevant information. The feature map including spatial direction information and channel feature information is then convolved with 3 × 3 DSC to boost the feature map’s integrated information quality. Finally, the eigenvalues are converged to yield the integrated feature information map *F_C_* by using the BN and Sigmoid activation function. In the short connection section, we also conduct 3 × 3 DSC on the original feature map information *F_O_* so that the attention map can update its weight under the approximation refinement feature. Then, execute feature fusion using *F_C_* to update the weights of the input feature map.

The preceding procedure is represented by the following equations:

$$F_{D}={\mathrm{DSC}}_{1\times 1}\left({\text{concat}}_{c}\left(\left[\text{Adap}-\text{AvgPool}\left(X_{c}\right),\text{Adap}-\text{AvgPool}\left(Y_{c}\right)\right]\right)\right)$$ (2)

$$F_{C}=\sigma \left(\mathrm{BN}\left({\mathrm{DSC}}_{3\times 3}\left({\mathrm{DSC}}_{1\times 1}\left(F_{\mathrm{Dx}}\right)⊙{\mathrm{DSC}}_{1\times 1}\left(F_{\mathrm{Dy}}\right)\right)\right)\right)$$ (3)

$$\mathrm{CA}=F_{C}⊙{\mathrm{DSC}}_{3\times 3}\left(F_{O}\right)$$ (4)

In Eq. 2, *X_c_* and *Y_c_* represent the horizontal and vertical tensors decomposed along the channel direction, *Adap* − *AvgPool* represents adaptive averaging pooling, *DSC*_1 × 1_ includes a 1 × 1 DSC, SN layer, and Serf activation function, and *concat_c_* represents stacking along the channel direction.

In Eq. 3, *F_Dx_* and *F_Dy_* represent the direction-aware feature map separated along the channel into vertical and horizontal tensors, BN is batch normalization, σ represents Sigmoid activation function, *DSC*_3 × 3_ represents a 3 × 3 DSC, and ⊙ represents pixel-by-pixel multiplication.

Equation 4 indicates the updated weight of the information in the input’s initial feature map.

In the Effectiveness of LRAM section, the experimental study of LRAM and the experimental comparisons of other attention mechanisms are discussed.

#### Proximity feature aggregation network with switchable atrous convolution and deconvolution

The color and texture features of tomato leaf diseases are extraordinarily intricate. Even if only 5 of these categories are detected in this study, there are still several issues. For instance, septoria leaf spot and bacterial spot are both grayish-brown, making their color channel features extremely similar. Not only do late blight and early blight have a similar dark brown color, but their textural features are also difficult to distinguish. If the feature fusion for such similarity information is inadequate, the nuanced information for distinguishing diseases will be lost. This impacts the performance of categorization during detection, which diminishes the accuracy of network predictions.

Feature pyramid networks (FPN) [34] is widely used in the feature fusion process for detectors and can greatly increase detection performance. It adopts a top route to jointly express features of different scales at different layers. This enables all prediction layers to contain rich semantic information, greatly enhancing the precision of multi-scale object detection. In YOLOX-S, PANet [35] introduces a bottom-up path to FPN. This shortens the information path between lower-level and higher-level features, allowing the entire feature structure to be improved with precise localization signals from lower levels. PANet has 2 information paths, but its transmission is unidirectional. For the entire backbone layer, neighboring layers share comparable features. We can employ nearby approximate information to strengthen the subtle characteristics of diseases and enhance the network’s ability to recognize minor differences, hence simplifying the distinction of subtle variances between disease categories.

Therefore, we modified PANet’s path transmission method. We replace the transmission method with proximity aggregation based on the 2 paths. We utilized the aforementioned recursive approach at the convolution level during the transfer of information between the higher and lower layers in order to highlight the tiny distinctions between diseases. In conclusion, SD-PFAN is proposed, and the structure is depicted in Fig. 3D. Compared with PANet, SD-PFAN multiplexes similar disease information at adjacent levels while transmitting bidirectional information. Its transmission method is bidirectional, with higher transmission efficiency and fusion times. This works well for distinguishing similar disease characteristics with lesser differences.

First, a 3 × 3 SAC [36] with a stride of 2 is employed in the downsampling process. Figure 3D shows the SAC structure. It convolves features with variable atrous rates and gathers the results with the help of a switching function. This module applies the mechanism of repeated observation and thinking, reuses similar features between neighboring levels, and emphasizes the tiny distinctions between diseases. SAC sets the rate of atrous convolution by using a 5 × 5 average pooling layer and a 1 × 1 convolution as a switching function [37]. The conversion equation is as follows:

$$\begin{array}{c} \text{Conv}(x,w,1)\overset{\text{Convert to SAC}}{\to }S(x)\cdot \text{Conv}(x,w,1)+\\ \left(1-S(x))\cdot \text{Conv}(x,w+\Delta w,r\right) \end{array}$$ (5)

where *x* is the input, *w* is the convolution operation’s weight, *r* is the SAC’s atrous rate, ∆*w* is a trainable weight, and the Switch function S(*x*) is composed of a 5 × 5 average pooling followed by a 1 × 1 convolution whose value is determined by the location and input information.

Second, in the upsampling process, we employ a 3 × 3 deconvolution [38] with a stride of 2. The deconvolution, unlike the interpolation-based method employed by YOLOX-S, offers learnable parameters. It can filter out unnecessary information, maintain disease characteristics consistent at multiple layers and has better feature mapping capabilities. Let *r_i_* be the expansion rate of the *i*th layer and *M_i_* be the maximum expansion rate of the *i*th layer, then the expanded convolution satisfies the following equation:

$$M_{i}=\text{max}[M_{i+1}-2^{*}r_{i},M_{i+1}-2^{*}(M_{i+1}-r_{i}),r_{i}]$$ (6)

Finally, SD-PFAN aggregates and multiplexes the feature information of neighboring layers along the channel dimension in order to improve information flow and feature fusion between different feature layers. This allows the output feature map to contain both shallow visual features and deep semantic features. Subsequent detection layers are supplemented with semantic information appropriate for classification and visual information appropriate for localization. Refer to the Effectiveness of SD-PFAN section for the combination selection analysis of SD-PFAN’s up- and downsampling components.

## Results

This section validates the model’s superiority by experimentally demonstrating that PLPNet overcomes the problems of similar environmental interference, intraclass variability, and interclass similarity in the tomato leaf disease detection challenge. Other subsections are divided into (a) describing the experimental environment and setup, including the hardware and software environments and training parameter settings; (b) evaluating the experimental performance measures of PLPNet; (c) analyzing the performance of PLPNet to verify the superiority of the model in this paper; (d) evaluating the effectiveness of each part of PLPNet and determining the role produced by each part; (e) conducting ablation experiments on the model; (f) comparison of PLPNet with other detectors to demonstrate PLPNet’s superiority over other methods in tomato leaf disease detection tasks; and (g) visualizing PLPNet detection results to intuitively evaluate PLPNet’s capability.

### Experimental environment and training details

All experiments in this study are completed in the same hardware and software environment to avoid differing experimental conditions from influencing PLPNet outcomes. The NVIDIA GeForce RTX 3060 GPU and AMD R7 5800H CPU were the primary hardware employed in this experiment. While Python, CUDA, and CUDNN versions have no effect on the experimental results, they must be compatible with the specific software and hardware. PLPNet was constructed using PyTorch 1.10.1 and MMDetection. Table 3 shows the specific hardware parameters.

**Table 3.** Software and hardware environment settings.

		
Hardware environment	CPU	AMD Ryzen 7 5800H with Radeon Graphics
	RAM	16 GB
	GPU	NVIDIA GeForce RTX 3060
	Video memory	6 GB
Software environment	OS	Windows 11
	CUDA Toolkit V11.3.1	
	CUDNN V8.2.1	
	mmdet 2.23.0	mmcv-full 1.4.8
	Python 3.7.11	
	Torch 1.10.1	Torchvision 0.11.2

The input images are 640 × 640 pixels in size. Before the input, the dataset was augmented by symmetric flipping, adding Gaussian noise (blur), and changing the brightness shading, resulting in a total of 13,597 images. In this study, we employ 10-fold cross-validation for training to properly evaluate the model’s performance while avoiding model overfitting. Thus, all input images are randomly divided into 10,877 training sets, 1,360 validation sets, and 1,360 test sets in an 8:1:1 ratio. The training set is used to train the model, the validation set is used to evaluate the performance and parameters of the training model and select the optimal model throughout the training process, and the test set is used to evaluate the model’s final performance.

In the training phase, this paper adopts the cosine annealing strategy to avoid the network from falling into the local optimum. This can help accelerate the model’s convergence and enhance stability. Experiments consist of 150 training epochs, with maximum and lowest learning rates of 1 × 10^−3^ and 1 × 10^−5^, respectively. The optimizer uses Adam, and the momentum is 0.9. Considering the performance of the hardware device and the training effect, the batch size is set to 8. The experimental settings are shown in Table 4.

**Table 4.** Experimental parameter settings.

Input size	640 × 640	Training strategy	Cosine
Maximum learning rate	1 × 10^−3^	Minimum learning rate	1 × 10^−5^
Optimizer	Adam	Momentum	0.9
Batch size	8	Number of iterations	150 epochs

### Evaluation indicators

In this paper, we evaluate the performance of the model by precision (P), recall (R), mAP, AR, FPS, parameter size, and GFLOPs.

The results are divided into 2 categories: *P* is the proportion of correct positive predictions to all positive predictions, and *R* represents the proportion of correct positive predictions to all positive actuals. The relevant formulas are as follows:

$$P=\frac{\mathrm{TP}}{\mathrm{TP}+\mathrm{FP}}\times 100\%$$ (7)

$$R=\frac{\mathrm{TP}}{\mathrm{TP}+\mathrm{FN}}\times 100\%$$ (8)

where *TP* is true positive, *FP* is false positive, *TN* is true negative, and *FN* is false negative.

*mAP* is an average measure that is commonly used in object detection to evaluate the performance of models with multiple classes. In this study, 5 different categories of tomato leaf diseases are studied, which may be referred to the assessment criteria. The formula for calculating *mAP* is as follows:

$$\mathrm{mAP}=\int_{0}^{1}P\left(r\right)\mathrm{dr}$$ (9)

The average recall (*AR*) is primarily used to assess the degree of model detection failure. The formula for calculating *AR* is as follows:

$$\mathrm{AR}=\frac{\text{Recall}}{n}$$ (10)

where *n* is the number of detected image frames.

Frames per second (*FPS*) is an important measure of detection speed. In this paper, it represents the average number of images detected per second. The formula for calculating *FPS* is as follows:

$$\mathrm{FPS}=\frac{1}{t}$$ (11)

where *t* is the time required to calculate each image.

Parameter size and GFLOPs are used to measure model size and complexity.

### PLPNet performance analysis

**Fig. 4.**
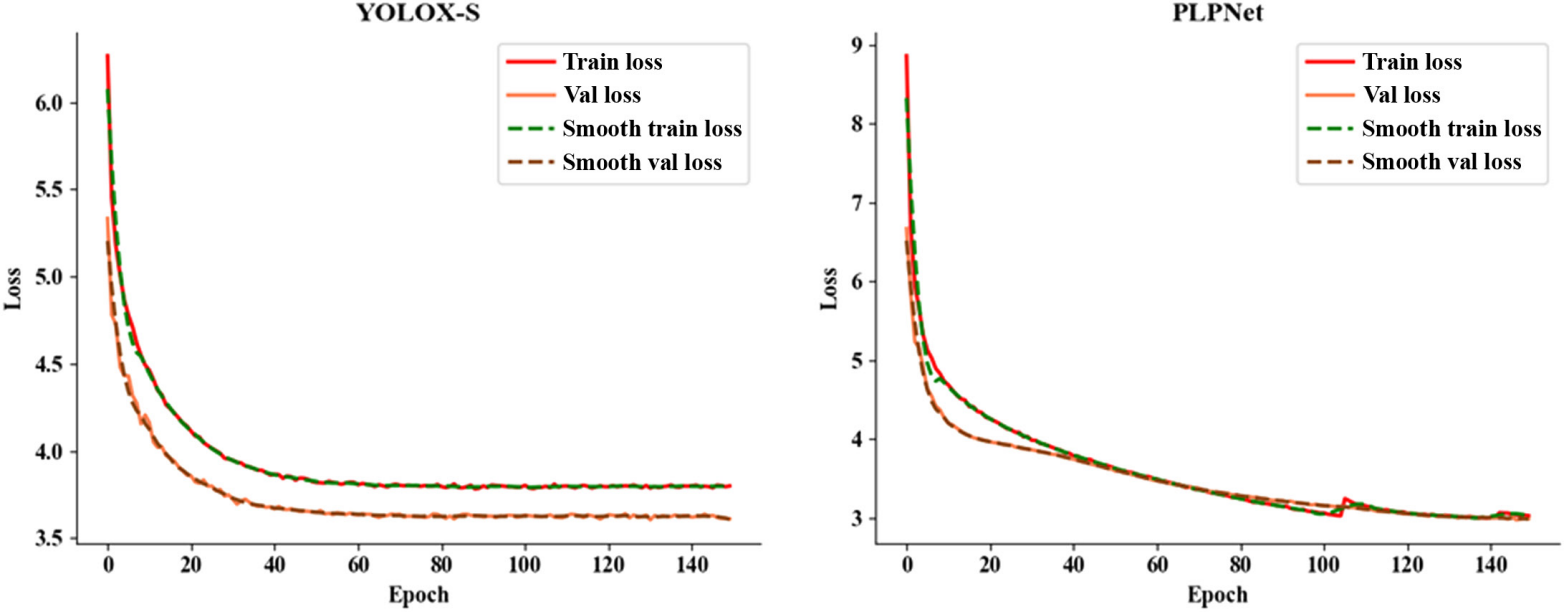
Loss variation of the training process.

To validate the effectiveness of PLPNet on YOLOX-S, we conduct a series of performance evaluation on the test set. To comprehensively analyze our network model, we conduct experimental evaluations on a number of measures, as shown in Table 5. The accuracy of PLPNet is improved considerably on both mAP and mAP50, partly because PLPNet has stronger feature extraction and feature fusion capabilities, and partly because the extra LRAM attention mechanism reduces information interference. We also evaluated the models’ AR values on 3 size areas (large, medium, and small), and the experimental results all demonstrated that PLPNet could detect more disease targets. Furthermore, we compared the differences between the 2 models during the training process, and their loss rate of change curves are shown in Fig. 4. As shown in the figure, the PLPNet model has lower loss function values at the convergence point and converges better. This demonstrates that the model matches the tomato leaf disease features better. In contrast, despite the fact that YOLOX-S degrades more rapidly throughout the training process, its fitness to the validation and training sets is biased. PLPNet enhances detection accuracy while maintaining basic detection speed, and the number of parameters in the model is equivalent to YOLOX-S.

**Table 5.** Comparison of model performance.

Method	YOLOX-S	PLPNet
mAP50	86.8	94.5
mAP	45.2	55.2
AR_L_	79.6	86.1
AR_M_	69.2	72.9
AR_S_	48.7	55.5
AR	46.8	54.4
FPS	36.81	25.45
Params (M)	8.968	10.633
GFLOPs (G)	26.806	39.405

### Module effectiveness experiments

This section contains and analyzes the evaluation metrics and parameters of PAC, LRAM, and SD-PFAN in detail. To correct for the effect of inconsistent data pre-processing methods on the studies, we adopt a mixed data augmentation strategy of mosaic and mixup for the input image data in our experiments. The results of the experiments are presented below.

#### Effectiveness of PAC

In this paper, we utilized PAC to replace the standard convolution of all CSP-Layer layers in the YOLOX-S backbone network to construct the PAC-CSPLayer, and the experimental results are shown in Table 6. The experimental results show that PAC can increase the accuracy of tomato leaf disease detection while introducing no more parameters than standard convolution in the CSPLayer. However, some speed is sacrificed. This is because the area mapped by the parallel structure of PAC is larger, and the efficiency of the algorithm decreases.

**Table 6.** Performance of PAC.

Method	mAP50	AR	FPS	Params (M)	GFLOPs (G)
YOLOX-S (CSPLayer)	86.8	46.8	36.81	8.968	26.806
+Replace Backbone CSPLayer conv	90.1	49.2	28.49	9.299	30.422
+Replace Backbone conv	90.9	50.5	25.23	9.415	32.835
+Replace prediction conv	91.0	50.4	20.21	9.624	35.315

Furthermore, we attempt to employ PAC in other parts of the network structure to further explore its effectiveness. The detection accuracy was marginally improved when the remaining standard convolution of backbone was replaced, but the inference time was increased. When the convolution in the prediction section was replaced further, the network’s accuracy did not improve, but the inference time grew considerably. This is because the anchor free strategy in the detection section has a similar effect to PAC’s weight adaptive computation. Taken together, it is clear that PAC is better suited for extracting features of tomato leaf diseases and that improving only the CSPLayer in the backbone network is the most cost-effective strategy.

#### Effectiveness of LRAM

In the Location reinforcement attention mechanism section, we mentioned the employment of SN and Serf to replace the traditional BN and ReLU. To validate the superiority of this combination, we evaluated the experimental results under different permutations, as shown in Table 7.

**Table 7.** Exploring the combination between normalization and activation functions.

Method	mAP50	AR
BN+ReLU	89.7	47.9
BN+Serf	89.9	48.2
SN+ReLU	90.3	48.7
SN+Serf	90.5	49.0

According to the experimental results, both SN and Serf were able to boost the module’s performance to some extent while without introducing an increasing number of parameters. They eliminate bias from different batch settings while preserving more disease information. This helps to increase precision. When compared to the standard BN + ReLU combination, the optimum SN + Serf combination raised mAP50 by 0.8% and AR by 1.1%.

To assess the overall performance of LRAM, we insert standard attention mechanisms such as SE, CBAM, CAM, and CA at the same position in the network. Table 8 shows the experimental results.

**Table 8.** Comparison of LRAM with other attention mechanisms.

Method	mAP50	AR	FPS	Param (M)	GFLOPs (G)
No improvement	86.8	46.8	36.81	8.968	26.806
SE	87.2	47.4	35.42	9.270	26.919
CBAM	87.9	48.1	37.54	9.342	27.211
CAM	88.3	48.2	33.60	9.558	27.839
CA	89.1	48.7	43.28	9.494	27.609
LRAM	90.5	49.0	39.87	9.722	28.148

The experimental results demonstrate that SE and CBAM can filter interference information and increase detection precision. However, the effect is modest. By locating the target region, the cross structure of CAM inhibits the expression of interference information, thus enhancing the detection precision. However, its positioning process is policy-driven and requires more calculation. CA has the highest performance, but the lowest precision compared to LRAM. This is detrimental to PLPNet since its pyramidal structure favors merging similar information. If similar noise information is not effectively filtered, it will add to the difficulty of fusion and produce confusing characteristics.

#### Effectiveness of SD-PFAN

SD-PFAN is composed of 3 major parts: the proximity feature aggregation network, deconvolution as upsampling, and SAC as downsampling. We compare PFAN and PANet on each combination to conduct experiments and study the performance of SD-PFAN in the feature fusion process and the optimal combination while employing SAC and deconvolution. Table 9 shows the study’s results.

**Table 9.** Exploring the optimal combination of SD-PFAN.

Method	mAP50	AR	FPS	Params (M)	GFLOPs (G)
YOLOX-S (PANet)	86.8	46.8	36.81	8.968	26.806
PFAN	87.9	47.0	53.45	8.857	26.533
PANet with deconvolution	87.2	46.9	39.84	9.291	28.758
PANet with SAC	88.1	47.3	37.59	9.357	29.012
PFAN with deconvolution	88.7	47.1	57.68	9.190	32.174
PFAN with SAC	89.2	47.8	55.34	9.256	33.537
PANet with deconvolution and SAC	88.5	47.7	42.46	9.680	35.964
PFAN with deconvolution and SAC	90.6	48.7	60.60	9.579	34.448

According to the experimental results, the PFAN structure is designed to be more lightweight and the aggregation of neighboring layers accelerates the model’s inference speed. The PFAN with SAC and deconvolution achieves the greatest improvement in accuracy when compared to the other 7 combinations, which effectively fuse similar tiny characteristics from different feature layers.

### Ablation experiments

We conducted ablation experiments on PLPNet to validate the effectiveness of the approach described in this paper, which is based on YOLOX-S, and the experimental results are shown in Table 10. We utilized the control variable method to sequentially add PAC, LRAM, and SD-PFAN and then conducted 8 groups of ablation experiments on these 3 modules in combination with PLPNet. When comparing groups G and H, it is demonstrated that PAC can perform superior feature extraction and greatly enhance model accuracy. When groups F and H are compared, it is demonstrated that LRAM filters the interference information in the model at the sacrifice of a small amount of speed. SD-PFAN has the best overall performance. It operates independently and provides excellent results in terms of speed and precision. In conclusion, PLPNet enhances mAP50 and AR by 7.7% and 7.6%, respectively, when compared to YOLOX-S, at the loss of a little bit of speed and without introducing a greater number of parameters. Meanwhile, the results of 8 sets of experiments show that PAC, LRAM, and SD-PFAN are all effective at enhancing model accuracy. Thus, PLPNet is superior to YOLOX-S for detecting tomato leaf disease.

**Table 10.** Ablation experiment of PLPNet.

Group	Method	mAP50	AR	FPS	Params (M)	GFLOPs (G)
A	YOLOX-S	86.8	46.8	36.81	8.968	26.806
B	A+PAC	90.1	49.2	28.49	9.299	30.422
C	A+LRAM	90.5	49.0	39.87	9.722	28.148
D	A+SD-PFAN	90.6	48.7	60.60	9.579	34.448
E	B+LRAM	92.8	52.0	23.30	10.052	31.764
F	B+SD-PFAN	93.0	51.9	31.35	9.909	38.063
G	C+SD-PFAN	91.6	49.3	43.29	10.332	35.789
H	E+SD-PFAN	94.5	54.4	25.84	10.633	39.405

### Comparing different models

We conducted comparison experiments with several traditional and current advanced object detection approaches on the same test environment and test set to further analyze PLPNet’s performance. Table 11 shows the test results.

**Table 11.** Compare the performance of PLPNet with other detectors.

Method	Backbone	mAP50	AR	FPS
Two-stage detectors				
Faster R-CNN	ResNet-101	72.6	35.5	–
R-FCN [42]	ResNet-101	71.7	36.8	–
CoupleNet [43]	ResNet-101	74.2	38.1	–
Faster R-CNN w TDM	Inception-ResNet-v2-TDM	76.6	41.4	–
Mask R-CNN [44]	ResNeXt-101	78.3	42.7	–
SINPER [45]	ResNet-101	85.4	45.5	–
Cascade R-CNN [46]	ResNet-101	87.5	47.3	–
MegDet [47]	ResNet-50	93.9	54.1	–
One-stage detectors				
SSD512	VGG-16	68.6	33.9	68.20
RetinaNet [48]	ResNeXt-101	73.5	35.7	75.88
RefineDet [49]	ResNet-101	74.8	36.2	40.39
CenterNet [50]	Hourglass-104	76.3	37.4	14.55
YOLOv3-ASFF [51]	Darknet-53	77.5	38.5	30.36
EfficientDet-D0 [52]	EfficientNet	79.3	40.7	17.22
NAS-FPN [53]	AmoebaNet	81.4	41.5	12.39
YOLOv4	CSPDarknet-53	79.9	41.3	33.62
YOLOv5-S	CSPDarknet-53	84.7	43.6	35.28
YOLOX-S	CSPDarknet-53	86.8	46.8	36.81
PLPNet	PAC-CSPDarknet-53	94.5	54.4	25.84

In the two-stage detector, R-CNN [39] initially generates 2,000 candidate boxes using selective search. Then, the pre-trained CNN model is employed to extract features from input images. Finally, support vector machine is utilized to finish the objective prediction. However, R-CNN needs feature extraction for each candidate frame, resulting in a large number of redundant features and a long detection time. SPPNet [40] introduces a spatial pyramid pool to transform the features corresponding to the candidate frame into fixed-length features. The method requires only a single extraction of features to generate features at multiple sizes. Despite SPPNet’s fast detection speed, the training process is still conducted in phases. Fast R-CNN [41] accelerates training and detection by integrating classification and bounding box regression training on the basis of the previous 2 methods. Faster R-CNN integrates candidate frame generation, classification, and regression into a single network for joint learning, which enhances training and detection speed but is less successful in detecting tiny objects. The Faster R-CNN with TDM, Mask R-CNN, and Cascade RCNN detectors are further optimized for Faster R-CNN in different aspects. According to the experimental results, PLPNet outperforms the majority of them in terms of accuracy when compared to a standard two-stage detector. While MegDet performs comparably to PLPNet in terms of accuracy, it is worse in terms of speed. This is definitely insufficient for real applications. It is also not as effective as PLPNet for detecting tomato leaf disease.

Among one-stage detectors, the vast speed advantage of standard SSD and RetinaNet demonstrates that one-stage detectors are more appropriate for real-world application environments. They are, however, insufficiently precise. This disadvantage has now been improved by the development of one-stage detectors. YOLOv3-ASFF and YOLOv4 achieved 77.5% and 79.9% on mAP50, respectively, and the FPS also satisfied the criteria of real-time detection. Furthermore, EfficientDet-D0 and NAS-FPN design neural networks using the approach of network structure search, which results in improved accuracy but raises inference time to some extent. YOLOv5-S, on the other hand, achieves good accuracy and speed results, whereas YOLOX-S has better performance on top of that, which is why we chose it as the primary reason for the baseline network. Our model exceeds YOLOX-S by 7.7% on mAP50, without much sacrifice in speed. PLPNet outperforms detectors from recent years in terms of overall performance. In particular, PLPNet, which is based on PAC and LRAM, was able to better extract the complicated characteristics of tomato leaf diseases and perform effective feature fusion utilizing SD-PFAN. Finally, the experiment’s accuracy achieved 94.5% while maintaining an FPS of 25.84. Although somewhat slower than YOLOX-S in terms of speed, it meets the real-time standards for tomato leaf disease detection.

PLPNet is the optimum model for detecting tomato leaf disease when compared to the other models in the table. We believe the following reasons as to why our PLPNet outperformed the other models in terms of performance: (a) PLPNet is improved based on YOLOX-S. It contains numerous advanced techniques to enhance the model’s accuracy and speed while also guaranteeing robust detection performance. (b) PAC is employed for the model’s backbone, which boosts the model’s capacity to extract complicated characteristics of tomato leaf diseases. (c) LRAM is added to the neck, which filters some interference information before the features enter fusion, enabling the feature map to express itself more clearly. (d) SD-PFAN strengthens the network’s feature fusion capability. This enables the model to reuse approximation characteristics and emphasize tiny variations in tomato leaf diseases. (e) The self-built dataset in this study eliminates several blurry and low-quality images, which benefits with model training.

### Comparison of visualization results

Table 12 shows how we visualized the detection results of YOLOX-S and PLPNet to better understand PLPNet. The detection frames, categories, and confidence are all presented on the detection results in the table. We utilized plant category abbreviations LB (late blight), EB (early blight), and SLs (septoria leaf spot) to better present the information of the detection frames.

**Table 12.** Comparison of visualization test results.

Method	Detection results		
YOLOX-S	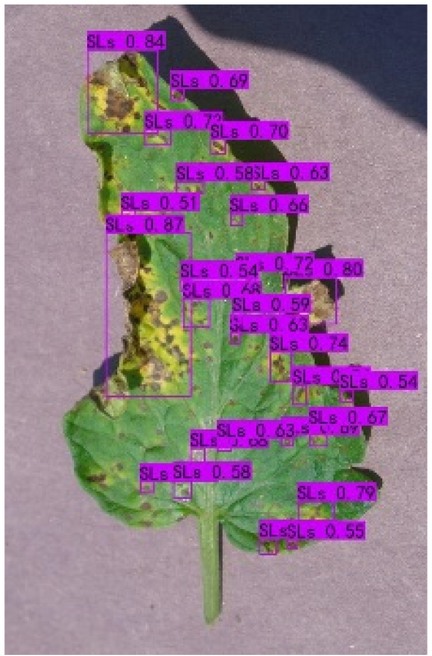	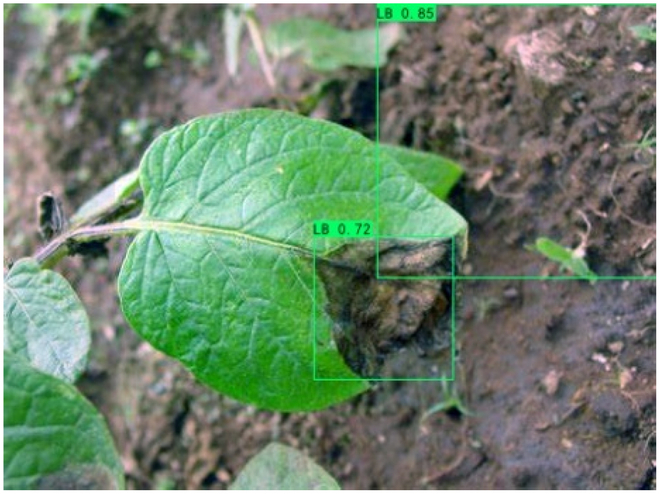	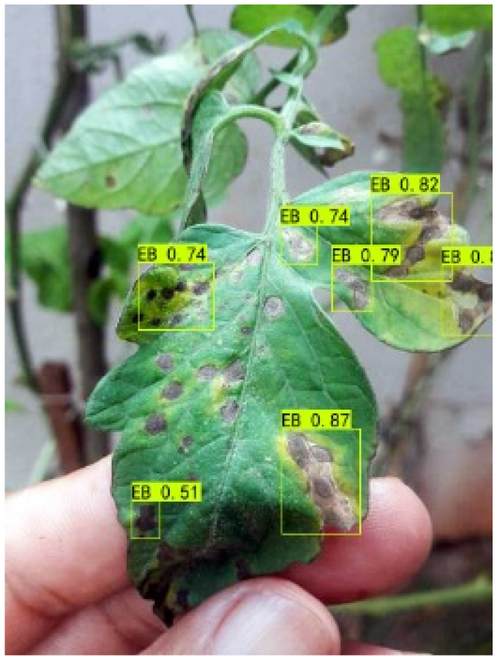
PAC	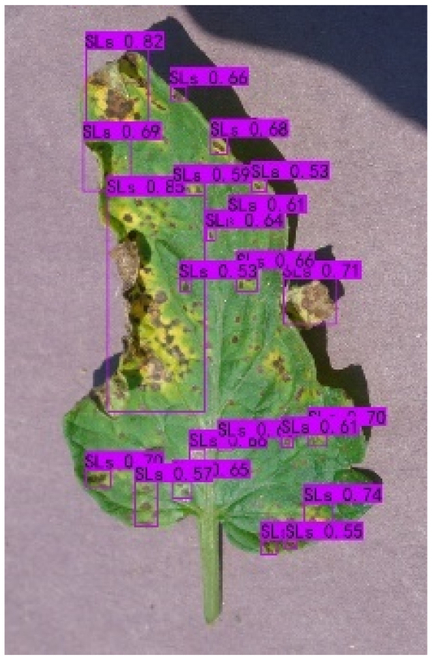	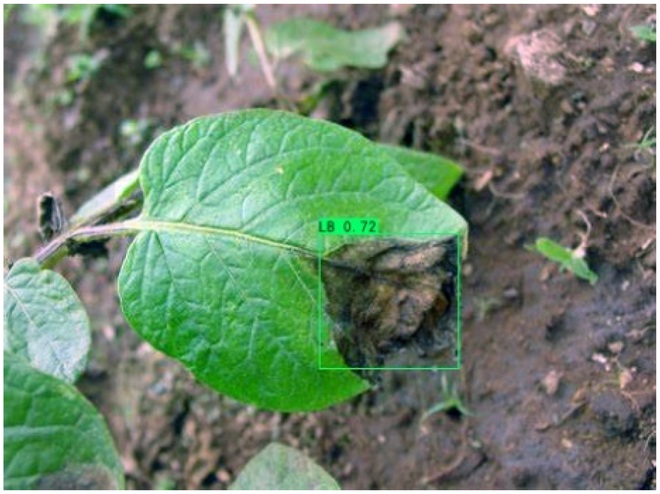	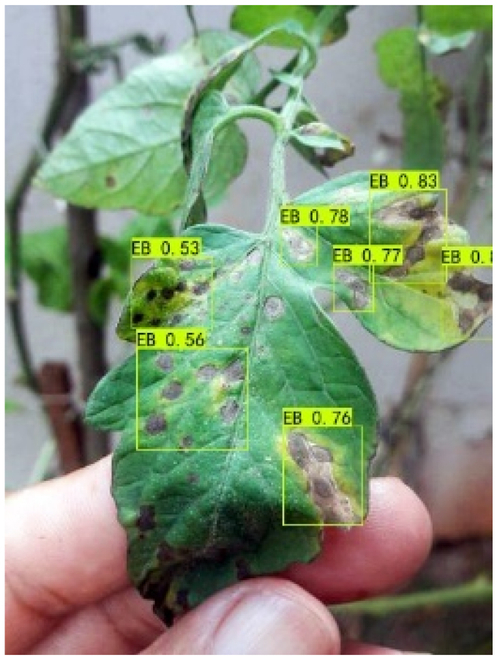
PAC+LRAM	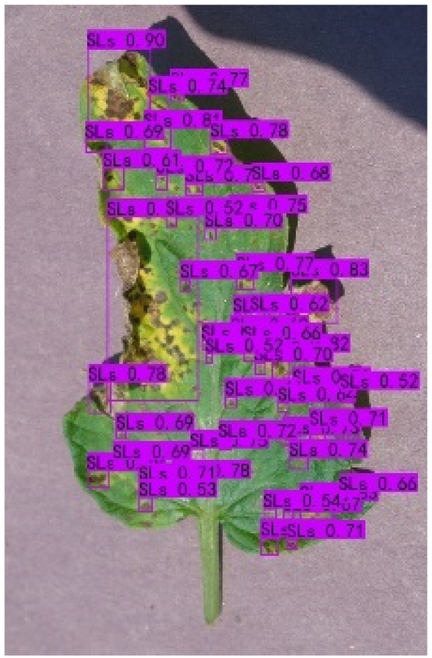	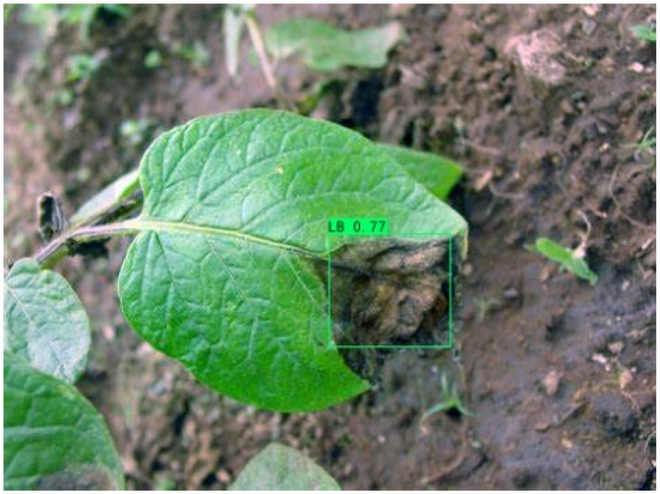	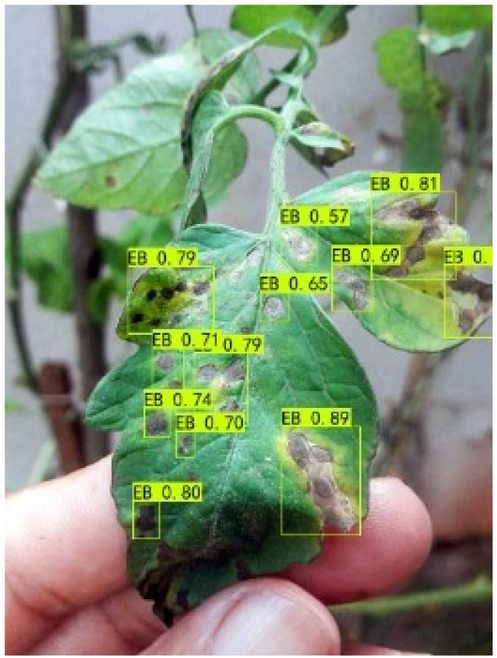
PLPNet	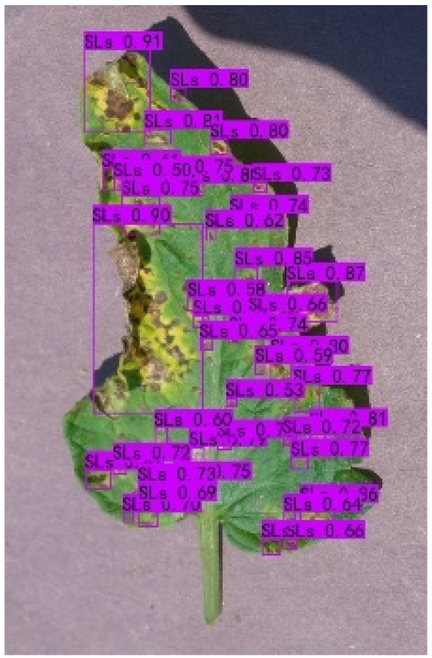	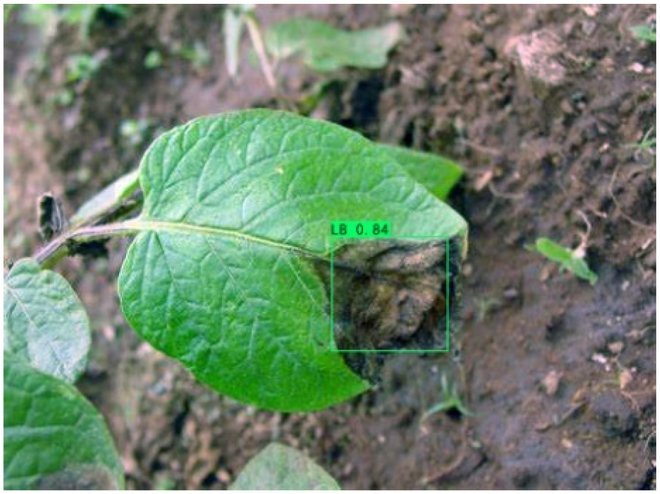	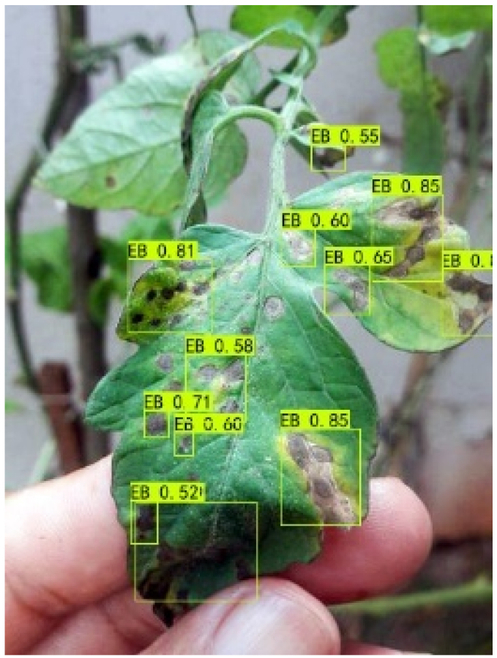
	A	B	C

In group A, we chose SLs with complicated internal disease characteristics to observe the detection outcomes. PLPNet, as compared to YOLOX-S, is capable of not only detecting more small-scale diseases but also catching larger-scale diseases. Furthermore, YOLOX-S does not detect several medium-sized diseases with complicated colors and textures. This is due to the multi-scale structure of PAC, which allows the network to have a more flexible perceptual field. This design reduces missed detection of small sizes, detects middle sizes more robustly, and enhances the integrity of large objects.

In group B, we chose a representative image of the soil backdrop nearby the disease area. The detection results show that YOLOX-S incorrectly and confidently treats a portion of the soil backdrop as the disease itself. In contrast, after utilizing PAC and LRAM, the network model not only learns to distinguish between the disease and the soil backdrop from a global perspective, but also suppresses the expression of irrelevant information to some extent. This filtered the noise for the SD-PFAN feature fusion that followed. The results of gradually rising confidence demonstrate the effectiveness of our approach.

In group C, we chose EB with similar characteristics to LB for the experiment to evaluate the network’s performance in detecting similar features. Both detectors did not appear to confuse the 2 types of diseases based on the detection results. However, when the objective was too dark or the texture expression was coarse, YOLOX-S did not perform as well as PLPNet. This is due to the fact that the structural design of SD-PFAN neighboring layer aggregation can emphasize the manifestation of tiny feature variations.

Furthermore, because PLPNet and YOLOX-S detected disease targets in all 3 sets of tests, our approach achieved greater degrees of confidence. However, both detectors missed a small number of targets, which may be related to the quality of the tomato leaf disease dataset during training. This will be the focus of our next phase of the study.

PLPNet outperforms YOLOX-S in terms of overall performance. It not only enhances YOLOX-accuracy but also optimizes its leakage and false detection problems.

## Discussion

**Fig. 5.**
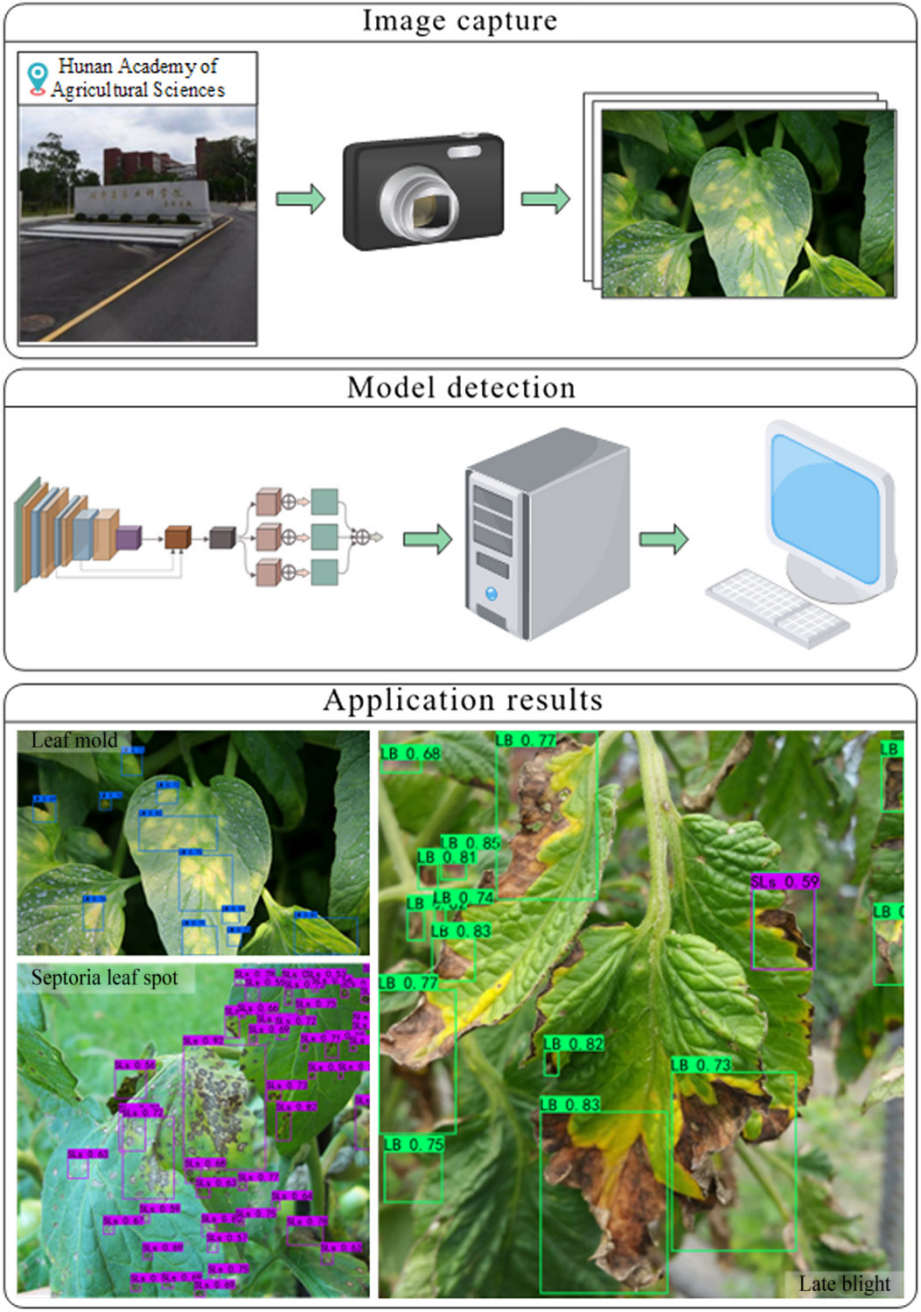
Workflow for a practical application.

From early March to late July 2022, this study monitored the occurrence of diseases at the Plant Protection Institute of Hunan Academy of Agricultural Sciences. We employed Canon cameras to gather 203 photos of the 5 categories of tomato leaf diseases detected in this study without impacting the environment. We utilized the images to test PLPNet and Fig. 5 shows the related workflow. First, we employed a Canon camera to capture images of several disease types in various environments. The photos were subsequently uploaded to the host computer for processing. Application results were ultimately presented on the monitor. Table 13 shows the results of a comparative test of PLPNet and YOLOX-S. In the Results section, we have discussed in detail the comparison of numerous metrics between these 2 models, as well as their similar FPS. Thus, we simply compare the confidence values in the detection results here.

**Table 13.** Compare YOLOX-S with PLPNet for practical applications.

Class	YOLOX-S	PLPNet
Late blight	0.84	0.87
Bacterial spot	0.79	0.83
Septoria leaf spot	0.75	0.83
Leaf mold	0.77	0.81
Early blight	0.83	0.86

The application results comparison data in the table show that the detection results of these 5 categories basically match the predicted improved effect. This demonstrates PLPNet’s superiority in detecting tomato leaf disease. In addition, we tested the real detection performance when several leaves are superimposed, as shown on the left side of the application results section in Fig. 5. The visualization results show that the performance of PLPNet in this environment does not vary massively. Despite a slight decrease in detection accuracy, there is no deviation in the location aspect. Because the training set comprises fewer images of the environment, the network’s prediction accuracy is reduced, but it still pays attention to these areas. This demonstrates PLPNet’s stability in detecting scenarios. However, there are certain unanticipated experimental results and examining these samples might give valuable insights for further network performance enhancement. Thus, in Fig. 5, we show a particular example to the right of the application results section. PLPNet detects multiple instances of late blight and septoria leaf spot in the same targeted area, which may be the consequence of disparate label assignment among classes in the dataset. The number of labels differed greatly among categories due to the numerous spot sizes produced by each disease. The original dataset had the greatest number of septoria leaf spots, and because the spots were mostly tiny targets, it had a substantial proportion of labels. PLPNet receives more feedback corrections of this class in backpropagation during the model’s training stage. Thus, the detector will be more inclined to consider the probability of that class during the detection stage, resulting in multiple prediction outcomes.

In a future study, we will consider adjusting the classification section of the network to balance the recognition probability of each class to alleviate the problem. In addition, the images are augmented by defining the comprehensive criteria based on the number of images and the number of disease labels to further ensure the dataset’s interclass balance.

## Conclusion

To explore the optimum approach for detecting tomato leaf disease, we propose a precise image-based tomato leaf disease detection approach using PLPNet. First, we proposed a PAC module to improve the model’s feature extraction capability for tomato leaf diseases. Then, LRAM is used to filter the noise in the feature information. Finally, in the process of feature fusion, SD-PFAN was designed to match the resemblance features of tomato leaf diseases. As a result, the approach overcomes the problem of leakage and misdetection in tomato leaf disease detection and enhances the model’s detection accuracy. More importantly, PLPNet improves the accuracy of the YOLOX model in the detection of tomato leaf disease and better matches the data features of tomato leaf disease images throughout the training process. This research can assist producers in detecting tomato leaf diseases in a timely and precise manner, as well as in making specific controls based on the kind of disease detected. This provides a new reference for deep learning in ensuring modern tomato agriculture. In the experimental section, we separated the collected 13,597 images into 8:1:1 training, validation, and test sets. PLPNet achieved 94.5% map50, 54.4% AR, and 25.84 FPS on this premise. PLPNet greatly enhances the accuracy of detection while maintaining the standard detection speed. Consequently, it outperforms other testing models and demonstrates the effectiveness of our enhanced approach.

Nonetheless, there are still issues that need to be resolved. The degree of grading of leaf diseases needs to be established to research disease early warning systems. The formation of leaf diseases is related to the parasitic process of bacteria, which is a local to global phase. Although the characteristics of different diseases differ, the same disease does not behave the same way in different environments. Detailing identifies the characteristics of the different disease phases and consequently defining the disease’s degree will not only offer early warning of disease onset but also allow for rapid remediation if the disease has already developed. As a result, both production losses and unnecessary resource waste can be eliminated. Furthermore, there are no visible signs when the disease first develops. It is impossible to determine whether or not they are affected based just on the images. In the future, we will need to merge Internet of Things sensor technology with deep learning to construct a disease infection process model to expand the comprehensiveness of disease early warning.

## Data Availability

Some of the datasets that were used and analyzed in this study have been uploaded to the website https://github.com/ZhouGuoXiong/PLPNet, and all the homemade datasets in this study (13,597 sheets in total) can be obtained by contacting the corresponding author.

## Supplementary Materials

Supplementary Materials Fig. S1. Problems with tomato leaf disease detection. Fig. S2. Examples of partial image labels. Fig. S3. The structure of PLPNet. Fig. S4. Loss variation of the training process. Fig. S5. Workflow for a practical application. Table S1. The number and characteristics of tomato leaves’ images. Table S2. Data augmentation type. Table S3. Software and hardware environment settings. Table S4. Experimental parameter settings. Table S5. Comparison of model performance. Table S6. Performance of PAC. Table S7. Explore the combination between normalization and activation functions. Table S8. Comparison of LRAM with other attention mechanisms. Table S9. Explore the optimal combination of SD-PFAN. Table S10. Ablation experiment of PLPNet. Table S11. Compare the performance of PLPNet with other detectors. Table S12. Comparison of visualization test results. Table S13. Compare YOLOX-S with PLPNet for practical applications.
