# Supplementary material for: A Precise Image-Based Tomato Leaf Disease Detection Approach Using PLPNet
Source: Plant Phenomics. 2023 May 12;5:0042. doi: 10.34133/plantphenomics.0042 (PMC10204740; doi:10.34133/plantphenomics.0042)
Supplement: Supplementary Materials — Fig. S1. Problems with tomato leaf disease detection. Fig. S2. Examples of partial image labels. Fig. S3. The structure of PLPNet. Fig. S4. Loss variation of the training process. Fig. S5. Workflow for a practical application. Table S1. The number and characteristics of tomato leaves’ images. Table S2. Data augmentation type. Table S3. Software and hardware environment settings. Table S4. Experimental parameter settings. Table S5. Comparison of model performance. Table S6. Performance of PAC. Table S7. Explore the combination between normalization and activation functions. Table S8. Comparison of LRAM with other attention mechanisms. Table S9. Explore the optimal combination of SD-PFAN. Table S10. Ablation experiment of PLPNet. Table S11. Compare the performance of PLPNet with other detectors. Table S12. Comparison of visualization test results. Table S13. Compare YOLOX-S with PLPNet for practical applications. [file plantphenomics.0042.f1.docx]

**Supplementary Materials**

**Figure 1. Problems with tomato leaf disease detection**

**Figure 2. Examples of partial image labels**

**Figure 3. The structure of PLPNet**

**Figure 4. Loss variation of the training process**

**Figure 5. Workflow for a practical application**

**Table 1 The number and characteristics of tomato leaves images**

| Category | Example | Characteristics | Number (before / after) | Proportion (before / after) |
| --- | --- | --- | --- | --- |
| Healthy | 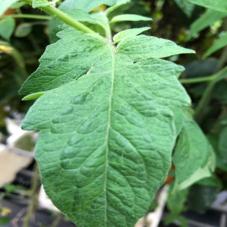 | The leaves are grassy green in color and texture, with no disease spots on the surface and distinct veins. | \ | \ |
| Bacterial spot | 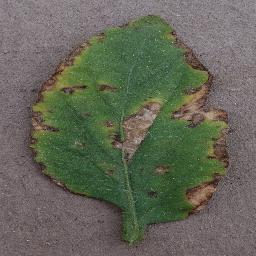 | The disease mostly damages the margins of the leaves, presenting gray-brown patches with a yellow halo surrounding them. | 1112/2708 | 20.47%/19.92% |
| Early Blight | 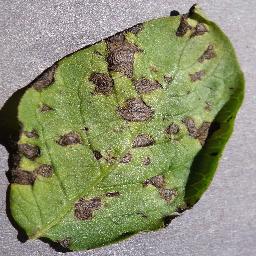 | The leaves generate little dark brown sub-circular spots, which are often linked to form larger irregular patches. | 841/2733 | 15.48%/20.10% |
| Late Blight | 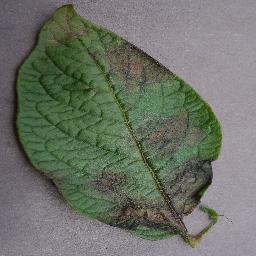 | The diseased area is a large area of dark brown water-soaked patchiness. | 1202/2705 | 22.12%/19.89% |
| Leaf Mold | 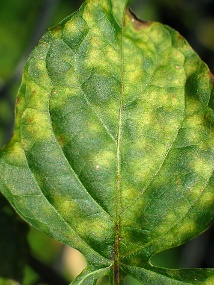 | The spots are yellow-green in hue and frequently cover a large area, filling nearly the whole leaf. | 881/2758 | 16.22%/20.28% |
| Septoria Leaf spot | 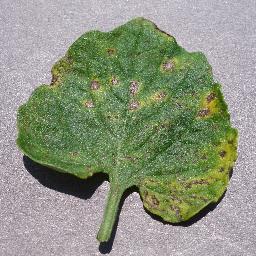 | The spots are gray-brown with a yellow halo, and the scale is often tiny, resembling a fish-eye appearance. | 1397/2693 | 25.71%/19.81% |

**Table 2 Data augmentation type**

| Horizontal flip | Gaussian blur | 20% brighter |
| --- | --- | --- |
| 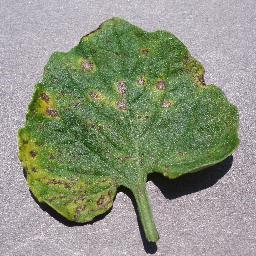 | 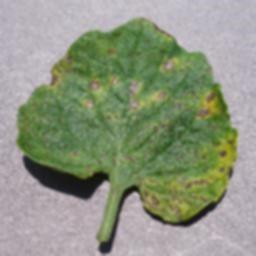 | 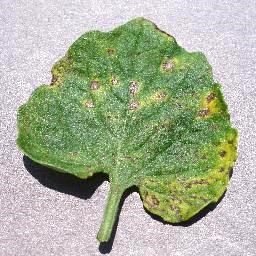 |
| Vertical flip | Gaussian noise | 20% less brightness |
| 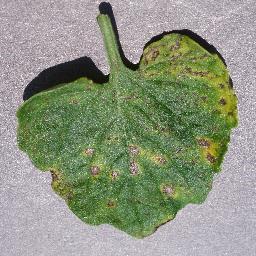 | 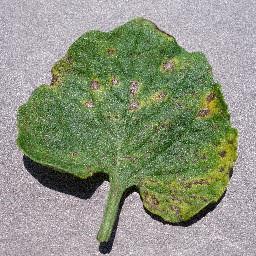 | 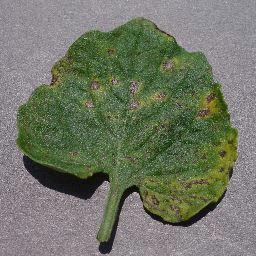 |

**Table 3 Software and hardware environment settings**

| Hardware  environment | CPU | AMD Ryzen 7 5800H with Radeon Graphics |
| --- | --- | --- |
|  | RAM | 16GB |
|  | GPU | NVIDIA GeForce RTX 3060 |
|  | Video Memory | 6GB |
| Software  Environment | OS | Windows 11 |
|  | CUDA Toolkit V11.3.1 |  |
|  | CUDNN V8.2.1 |  |
|  | mmdet 2.23.0 | mmcv-full 1.4.8 |
|  | Python 3.7.11 |  |
|  | Torch 1.10.1 | Torchvision 0.11.2 |

**Table 4 Experimental parameter settings**

| Input size | $640\times640$ | Training strategy | Cosine |
| --- | --- | --- | --- |
| Maximum learning rate | $1\times{10}^{-3}$ | Minimum learning rate | $1\times{10}^{-5}$ |
| optimizer | Adam | momentum | 0.9 |
| Batchsize | 8 | Number of iterations | 150 epochs |

**Table 5 Comparison of model performance**

| Method | YOLOX-S | PLPNet |
| --- | --- | --- |
| mAP50 | 86.8 | 94.5 |
| mAP | 45.2 | 55.2 |
| AR_L_ | 79.6 | 86.1 |
| AR_M_ | 69.2 | 72.9 |
| AR_S_ | 48.7 | 55.5 |
| AR | 46.8 | 54.4 |
| FPS | 36.81 | 25.45 |
| Params (M) | 8.968 | 10.633 |
| GFLOPs (G) | 26.806 | 39.405 |

**Table 6 Performance of PAC**

| Method | mAP50 | AR | FPS | Params (M) | GFLOPs (G) |
| --- | --- | --- | --- | --- | --- |
| YOLOX-S (CSPLayer) | 86.8 | 46.8 | 36.81 | 8.968 | 26.806 |
| +Replace Backbone CSPLayer conv | 90.1 | 49.2 | 28.49 | 9.299 | 30.422 |
| +Replace Backbone conv | 90.9 | 50.5 | 25.23 | 9.415 | 32.835 |
| +Replace prediction conv | 91.0 | 50.4 | 20.21 | 9.624 | 35.315 |

**Table 7 Explore the combination between normalization and activation functions.**

| Method | mAP50 | AR |
| --- | --- | --- |
| BN+ReLU | 89.7 | 47.9 |
| BN+Serf | 89.9 | 48.2 |
| SN+ReLU | 90.3 | 48.7 |
| SN+Serf | 90.5 | 49.0 |

**Table 8 Comparison of LRAM with other attention mechanisms**

| Method | mAP50 | AR | FPS | Param (M) | GFLOPs (G) |
| --- | --- | --- | --- | --- | --- |
| No improvement | 86.8 | 46.8 | 36.81 | 8.968 | 26.806 |
| SE | 87.2 | 47.4 | 35.42 | 9.270 | 26.919 |
| CBAM | 87.9 | 48.1 | 37.54 | 9.342 | 27.211 |
| CAM | 88.3 | 48.2 | 33.60 | 9.558 | 27.839 |
| CA | 89.1 | 48.7 | 43.28 | 9.494 | 27.609 |
| LRAM | 90.5 | 49.0 | 39.87 | 9.722 | 28.148 |

**Table 9 Explore the optimal combination of SD-PFAN**

| Method | mAP50 | AR | FPS | Params (M) | GFLOPs (G) |
| --- | --- | --- | --- | --- | --- |
| YOLOX-S (PANet) | 86.8 | 46.8 | 36.81 | 8.968 | 26.806 |
| PFAN | 87.9 | 47.0 | 53.45 | 8.857 | 26.533 |
| PANet with Deconvolution | 87.2 | 46.9 | 39.84 | 9.291 | 28.758 |
| PANet with SAC | 88.1 | 47.3 | 37.59 | 9.357 | 29.012 |
| PFAN with Deconvolution | 88.7 | 47.1 | 57.68 | 9.190 | 32.174 |
| PFAN with SAC | 89.2 | 47.8 | 55.34 | 9.256 | 33.537 |
| PANet with Deconvolution and SAC | 88.5 | 47.7 | 42.46 | 9.680 | 35.964 |
| PFAN with Deconvolution and SAC | 90.6 | 48.7 | 60.60 | 9.579 | 34.448 |

**Table 10 Ablation experiment of PLPNet.**

| Group | Method | mAP50 | AR | FPS | Params (M) | GFLOPs (G) |
| --- | --- | --- | --- | --- | --- | --- |
| A | YOLOX-S | 86.8 | 46.8 | 36.81 | 8.968 | 26.806 |
| B | A+PAC | 90.1 | 49.2 | 28.49 | 9.299 | 30.422 |
| C | A+LRAM | 90.5 | 49.0 | 39.87 | 9.722 | 28.148 |
| D | A+SD-PFAN | 90.6 | 48.7 | 60.60 | 9.579 | 34.448 |
| E | B+LRAM | 92.8 | 52.0 | 23.30 | 10.052 | 31.764 |
| F | B+SD-PFAN | 93.0 | 51.9 | 31.35 | 9.909 | 38.063 |
| G | C+SD-PFAN | 91.6 | 49.3 | 43.29 | 10.332 | 35.789 |
| H | E+SD-PFAN | 94.5 | 54.4 | 25.84 | 10.633 | 39.405 |

**Table 11 Compare the performance of PLPNet with other detectors.**

| Method | Backbone | mAP50 | AR | FPS |
| --- | --- | --- | --- | --- |
| Two-stage detectors | | | | |
| Faster R-CNN | ResNet-101 | 72.6 | 35.5 | \ |
| R-FCN [39] | ResNet-101 | 71.7 | 36.8 | \ |
| CoupleNet [40] | ResNet-101 | 74.2 | 38.1 | \ |
| Faster R-CNN w TDM | Inception-ResNet-v2-TDM | 76.6 | 41.4 | \ |
| Mask R-CNN [41] | ResNeXt-101 | 78.3 | 42.7 | \ |
| SINPER [42] | ResNet-101 | 85.4 | 45.5 | \ |
| Cascade R-CNN [43] | ResNet-101 | 87.5 | 47.3 | \ |
| MegDet [44] | ResNet-50 | 93.9 | 54.1 | \ |
| One-stage detectors | | | | |
| SSD512 | VGG-16 | 68.6 | 33.9 | 68.20 |
| RetinaNet [45] | ResNeXt-101 | 73.5 | 35.7 | 75.88 |
| RefineDet [46] | ResNet-101 | 74.8 | 36.2 | 40.39 |
| CenterNet [47] | Hourglass-104 | 76.3 | 37.4 | 14.55 |
| YOLOv3-ASFF [48] | Darknet-53 | 77.5 | 38.5 | 30.36 |
| EfficientDet-D0 [49] | EfficientNet | 79.3 | 40.7 | 17.22 |
| NAS-FPN [50] | AmoebaNet | 81.4 | 41.5 | 12.39 |
| YOLOv4 | CSPDarknet-53 | 79.9 | 41.3 | 33.62 |
| YOLOv5-S | CSPDarknet-53 | 84.7 | 43.6 | 35.28 |
| YOLOX-S | CSPDarknet-53 | 86.8 | 46.8 | 36.81 |
| PLPNet | PAC-CSPDarknet-53 | 94.5 | 54.4 | 25.84 |

**Table 12 Comparison of visualization test results**

| Method | Detection results | | |
| --- | --- | --- | --- |
| YOLOX-S | 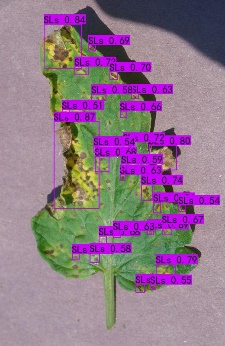 | 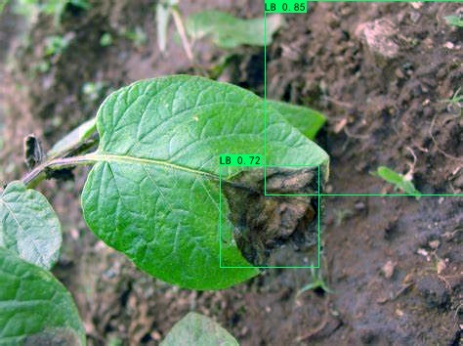 | 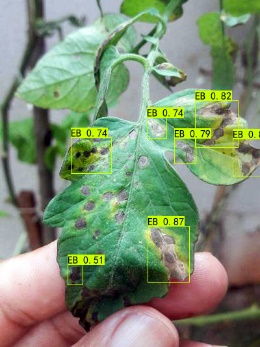 |
| PAC | 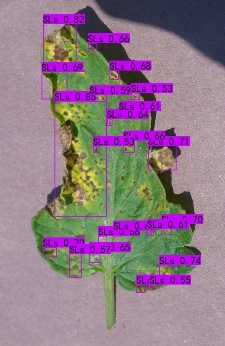 | 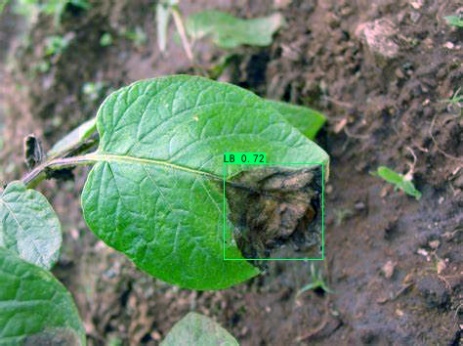 | 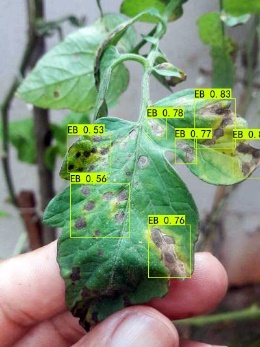 |
| PAC+LRAM | 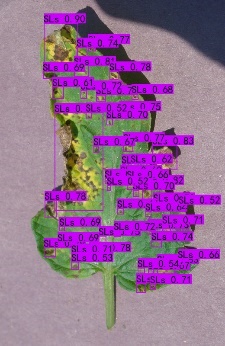 | 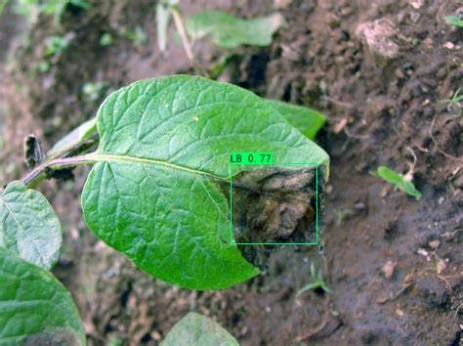 | 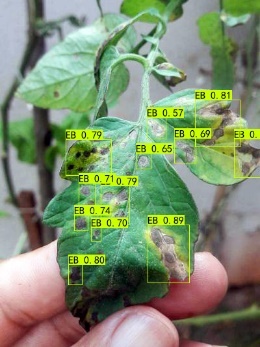 |
| PLPNet | 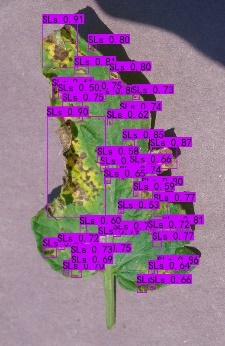 | 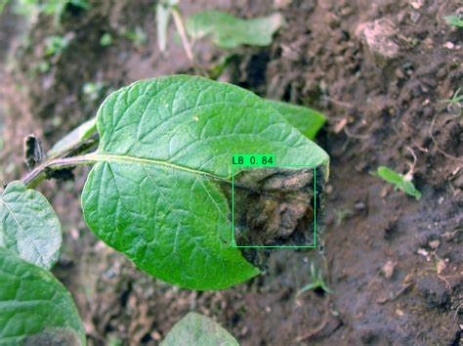 | 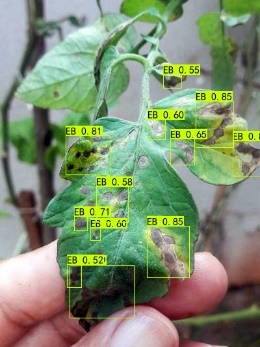 |
|  | (a) | (b) | (c) |

**Table 13 Compare YOLOX-S with PLPNet for practical applications.**

| Class | YOLOX-S | PLPNet |
| --- | --- | --- |
| Late blight | 0.84 | 0.87 |
| Bacterial spot | 0.79 | 0.83 |
| Septoria leaf spot | 0.75 | 0.83 |
| Leaf mold | 0.77 | 0.81 |
| Early blight | 0.83 | 0.86 |
